# Platelets Guide Leukocytes to Their Sites of Extravasation

**DOI:** 10.1371/journal.pbio.1002459

**Published:** 2016-05-06

**Authors:** Gabriele Zuchtriegel, Bernd Uhl, Daniel Puhr-Westerheide, Michaela Pörnbacher, Kirsten Lauber, Fritz Krombach, Christoph Andreas Reichel

**Affiliations:** 1 Department of Otorhinolaryngology, Head and Neck Surgery, Klinikum der Universität München, Munich, Germany; 2 Walter Brendel Centre of Experimental Medicine, Klinikum der Universität München, Munich, Germany; 3 Department of Radiation Oncology, Klinikum der Universität München, Munich, Germany; National Cancer Institute, UNITED STATES

## Abstract

Effective immune responses require the directed migration of leukocytes from the vasculature to the site of injury or infection. How immune cells “find” their site of extravasation remains largely obscure. Here, we identified a previously unrecognized role of platelets as pathfinders guiding leukocytes to their exit points in the microvasculature: upon onset of inflammation, circulating platelets were found to immediately adhere at distinct sites in venular microvessels enabling these cellular blood components to capture neutrophils and, in turn, inflammatory monocytes via CD40-CD40L-dependent interactions. In this cellular crosstalk, ligation of PSGL-1 by P-selectin leads to ERK1/2 MAPK-dependent conformational changes of leukocyte integrins, which promote the successive extravasation of neutrophils and monocytes to the perivascular tissue. Conversely, blockade of this cellular partnership resulted in misguided, inefficient leukocyte responses. Our experimental data uncover a platelet-directed, spatiotemporally organized, multicellular crosstalk that is essential for effective trafficking of leukocytes to the site of inflammation.

## Abstract 2

This study identifies a previously unanticipated role for platelets as pathfinders, guiding leukocytes to the sites at which they can exit the microvasculature; this process appears to be critical for an effective immune response.

## Author Summary

White blood cells (leukocytes) are the effector cells of the immune system. The movement (extravasation) of leukocytes from the bloodstream to the surrounding tissue is a prerequisite for proper host defense. Platelets are anucleate cell particles that circulate in the blood and play a fundamental role in hemostasis. Here, we report a previously unrecognized function of platelets as "pathfinders" guiding leukocytes to their site of extravasation. Upon onset of the inflammatory response, platelets were found to immediately adhere to specific sites in the smallest venular microvessels. At these "hot spots", platelets capture intravascularly crawling neutrophils and, in turn, inflammatory monocytes. The cellular crosstalk arising from these interactions leads to conformational changes of distinct adhesion molecules on the surface of leukocytes, subsequently promoting the extravasation of these immune cells to the inflamed tissue. Conversely, blockade of this cellular partnership leads to misguided and inefficient leukocyte responses. Thus, platelet-directed guidance of leukocytes to confined sites of extravasation appears to be a critical step in the recruitment process of immune cells, which might emerge as a promising therapeutic target for the prevention and treatment of inflammatory pathologies.

## Introduction

Directed migration of leukocytes from the vasculature to the site of injury or infection is a prerequisite for effective immune responses. Neutrophils are the first immune cells infiltrating the inflamed tissue, followed by a second wave of inflammatory monocytes (iMOs) amplifying the inflammatory reaction [1].

To enter the site of inflammation, leukocytes “roll” on the luminal surface of microvascular endothelial cells before these immune cells stabilize their interactions and adhere to the inner vessel wall. Extensive signaling between arrested leukocytes and the endothelium then triggers adhesion strengthening and leads to intraluminal crawling of these blood cells along the microvasculature in search for suitable sites of extravasation. Subsequently, leukocytes squeeze between adjacent endothelial cells, penetrate the perivenular basement membrane, and subendothelially locomote to gaps between pericytes from where they finally migrate into the interstitial tissue [2–6]. Whereas the basic principles of this multistep cascade have been characterized in the past decades, it remains poorly understood how leukocytes can “find” their sites of extravasation.

Platelets are anucleated cell particles released from bone marrow megakaryocytes into the circulation [7]. In addition to their fundamental role in hemostasis, platelets are increasingly recognized as participants in different biological processes including immune responses [8–14]. With respect to the capability of these cells to establish interactions with leukocytes and endothelial cells [15–19], we hypothesized that platelets guide leukocytes to their site of extravasation.

Here, we identified a previously unrecognized role of platelets as pathfinders navigating leukocytes to their exit points in the inflamed microvasculature: upon onset of inflammation, platelets immediately adhere at endothelial junctions in the smallest venular microvessels and capture neutrophils via CD40-CD40L/CD154-dependent interactions. Intravascularly adherent platelets and neutrophils, in turn, cooperatively recruit iMOs to these “hot spots” through a CD40-, CD40L/CD154-, and L-selectin/CD62L-mediated intraluminal interplay of these blood cells. In this cellular crosstalk, ligation of PSGL-1 by P-selectin/CD62P induces ERK1/2 MAPK-dependent conformational changes of surface-expressed leukocyte integrins. Together with ICAM-1/CD54, ICAM-2/CD102, VCAM-1/CD106, PECAM-1/CD31, and JAM-A, activated integrins subsequently promote the successive extravasation of neutrophils and iMOs to the perivascular tissue. Conversely, blockade of this cellular partnership leads to misguided, inefficient leukocyte responses collectively uncovering a platelet-directed, spatiotemporally organized, multicellular crosstalk that is essential for effective trafficking of leukocytes to the site of inflammation.

## Results

### Interactions between Platelets, Myeloid Leukocytes, and Endothelial Cells in the Inflamed Microvasculature

**Fig 1:**
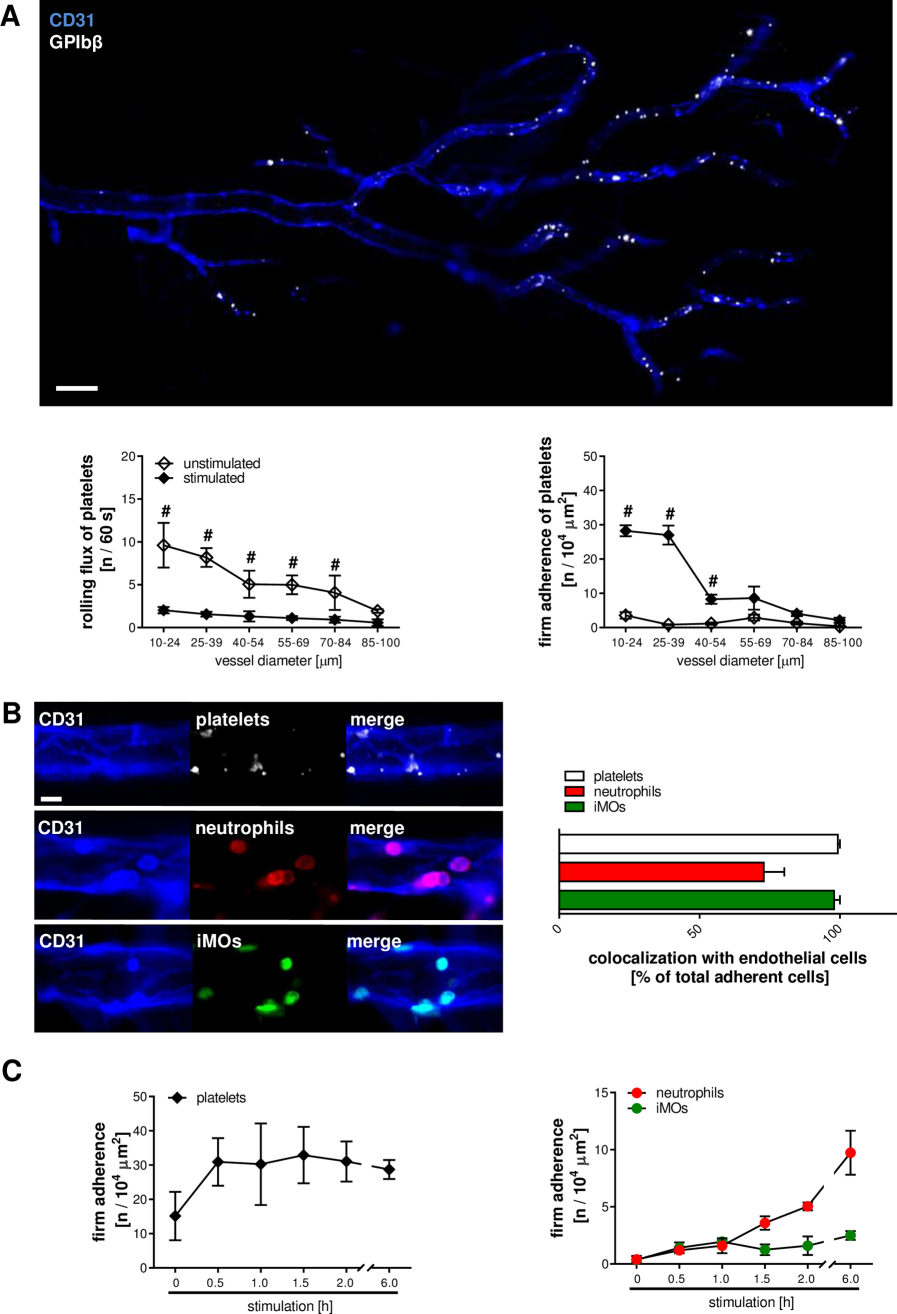
Endothelial cell interactions of platelets in the microvasculature. Interactions of platelets (white) and endothelial cells (blue) were analyzed in the microvasculature of the inflamed cremaster muscle of CX_3_CR1^GFP/+^ mice by multichannel in vivo microscopy (**A**; scale bar 100 μm). Panels show results for intravascularly rolling and firmly adherent platelets in venules after 6 h of intrascrotal stimulation with phosphate-buffered saline (PBS) or CCL2 in dependency of the venular diameter (mean ± standard error of the mean [SEM] for *n* = 4 per group; #*p* < 0.05 versus PBS). Panel (**B;** scale bar: 10 μm) shows the relative localization of platelets (white), neutrophils (red), and iMOs (green) to PECAM-1/CD31-immunoreactive endothelial junctions (blue; mean ± SEM for *n* = 3 per group). Panel (**C**) shows the adhesion dynamics of platelets, neutrophils, and iMOs during the course of the acute inflammatory response (mean ± SEM for *n* = 3–4 per group).

In a first approach, we sought to identify the individual sites that are utilized by neutrophils and iMOs to extravasate into the perivascular tissue. For this purpose, endothelial cell interactions of these immune cells were analyzed in the inflamed microvasculature of the cremaster muscle of “monocyte-reporter mice” (CX_3_CR1^GFP/+^ mice; exhibiting fluorescence-labeled monocytes as well as NK cells and T cell subsets [20]) by using multichannel in vivo microscopy (**S1 Video**). Classical/inflammatory (GFP^low^ leukocytes) and nonclassical monocytes (ncMOs; GFP^high^ leukocytes) in CXC_3_CR1^GFP/+^ mice were differentiated by their relative fluorescence intensity as described previously [21,22]. This experimental approach was validated by in vivo immunostaining with fluorescence-labeled monoclonal anti-Ly6C antibodies demonstrating strong expression of Ly-6C on GFP^low^ leukocytes (classical/iMOs) and the absence of expression of Ly-6C on GFP^high^ leukocytes (ncMOs; **S1A Fig**). In the acute inflammatory response, only about 3% of intravascularly rolling GFP-positive leukocytes were NK1.1-positive NK cells or CD3-positive T cells, and approximately 2% of intravascularly adherent GFP-positive leukocytes were positive for NK1.1 or CD3. These data collectively indicate that in postcapillary venules of the acutely inflamed cremaster muscle of CX_3_CR1^GFP/+^ mice endothelial cell interactions of GFP-positive leukocytes almost exclusively represent monocyte responses (**S1B Fig**). In addition, interactions of platelets (visualized by a fluorescence-labeled anti-GPIbβ antibody) and neutrophils (visualized by a fluorescence-labeled anti-Ly-6G antibody) with endothelial cells were examined in our in vivo microscopy experiments (**Fig 1A and 1B**).

Cytokines such as tumor necrosis factor (TNF), interleukin-1β (IL-1β), or interferon-γ (IFN-γ), and lipid mediators, predominantly elicit neutrophil (but barely monocyte) extravasation in the initial inflammatory response (<6 h; **S2 and S3 Figs**) [22]. To enable us to study the involvement of monocytes under acute inflammatory conditions, however, the C-C motif chemokine CCL2 was used as principal inflammatory stimulus in our study. This chemokine potently induces extravasation of both neutrophils and iMOs in a variety of inflammatory disorders [23,24]. In this context, it has been shown that CCL2 causes the degranulation of tissue mast cells and induces the synthesis of inflammatory mediators including leukotrienes and platelet activating factor [25,26]. This leads to an increase in microvascular permeability and causes enhanced expression of adhesion/signaling molecules on the surface of microvascular endothelial cells ultimately resulting in a massive leukocyte infiltration of the perivascular tissue [22,25,26].

To proof our key findings under different inflammatory conditions, selected experiments were repeated using the cytokines TNF, IL-1β, or IFN-γ as inflammatory stimulus (**S2 and S3 Figs**). To ensure a broad affection of the tissue by the inflammatory response, the inflammatory mediators were applied intrascrotally prior to in vivo microscopy.

In unstimulated control animals, some rolling, but only few intravascularly adherent, platelets were detected in cremasteric postcapillary venules exhibiting inner diameters of less than 40 μm. Upon stimulation with CCL2 or TNF, however, the number of intravascularly adherent platelets immediately increased, whereas the number of rolling platelets significantly decreased in this vessel segment (**Fig 1A; S2A Fig**). In detail, more than 95% of firmly adherent platelets were found to colocalize with PECAM-1/CD31-immunoreactive endothelial cell junctions (**Fig 1B; S1 Video**). Moreover, we observed platelets to be the first cellular blood components arresting at the inflamed endothelium (within 30 min after onset of inflammation with CCL2 or TNF), whereas intravascular adherence of neutrophils (>60 min after onset of inflammation elicited by CCL2 or TNF) and iMOs (>180 min after onset of inflammation; upon stimulation with CCL2, but not with TNF) occurred at later time points (**Fig 1C; S2B Fig**). About 80% of firmly adherent Ly6G-positive neutrophils and more than 90% of firmly adherent iMOs (represented by GFP^low^ cells) were directly attached to endothelial cell junctions (**Fig 1B; S1 Video**).

Remarkably, in postcapillary venules with vessel diameters of more than 40 μm, only low numbers of platelets interacted with the endothelium upon stimulation with CCL2 or TNF (**Fig 1A; S2A Fig**), whereas platelet–endothelial cell interactions were virtually absent in arterioles.

**Fig 2:**
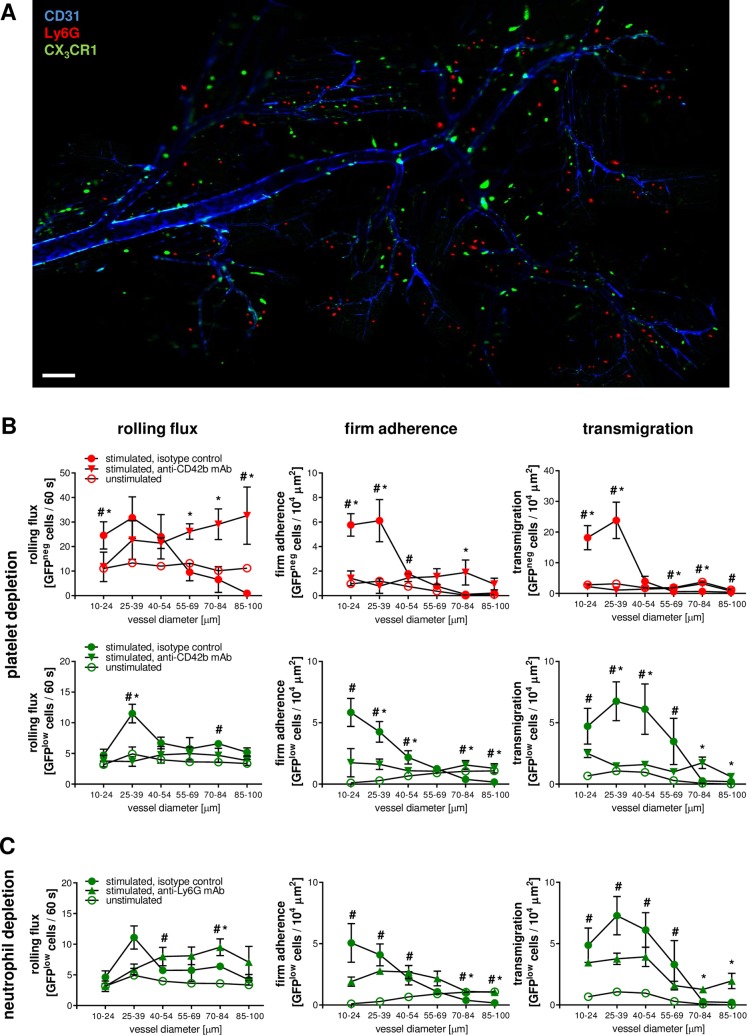
Interactions of platelets, neutrophils, and iMOs in the microvasculature. Interactions of neutrophils (red) and iMOs (green) with endothelial cells (blue) were analyzed in the microvasculature of the cremaster muscle of CX_3_CR1^GFP/+^ mice by multichannel in vivo microscopy (**A**; scale bar 100 μm). Panels show results for intravascularly rolling and firmly adherent as well as transmigrated neutrophils or iMOs in venules after 6 h of intrascrotal stimulation with PBS or CCL2 receiving a platelet-depleting anti-CD42b mAb (**B**), a neutrophil-depleting anti-Ly-6G mAb (**C**), or isotype control antibodies in dependency of the venular diameter (mean ± SEM for *n* = 4 per group; #*p* < 0.05 versus PBS; *p < 0.05 versus isotype control).

Similar to platelets, endothelial cell interactions and transmigration events of neutrophils (upon stimulation with CCL2, TNF, IL-1β, or IFN-γ) and iMOs (only upon stimulation with CCL2 or IFN-γ) in the inflamed microvasculature were predominantly observed in postcapillary venules exhibiting an inner diameter of approximately 25 μm, but not in arterioles. Interestingly, antibody-mediated depletion of platelets almost completely abolished intravascular adherence and subsequent transmigration of neutrophils (upon stimulation with CCL2, TNF, IL-1β, or IFN-γ) and iMOs (only upon stimulation with CCL2 or IFN-γ) in this venular vessel segment, whereas leukocyte rolling was consecutively enhanced in larger venules exhibiting diameters of more than 40 μm (**Fig 2A and 2B; S2C and S3 Figs**). In contrast, the proportion of intravascularly crawling neutrophils and monocytes to total adherent neutrophils or monocytes in response to CCL2 remained unaltered upon platelet depletion (**S4 Fig**). Furthermore, depletion of neutrophils resulted in a shift of endothelial cell interactions and transmigration events of iMOs from smaller (<40 μm diameter) to larger diameter (>40 μm diameter) segments of postcapillary venules upon stimulation with CCL2 (**Fig 2C**).

Depletion of platelets or neutrophils (by 95% compared with controls; **S1 Table**) was confirmed in peripheral blood samples.

### Endothelial Expression of Adhesion and Signaling Molecules, Composition of the Vascular Wall, and Shear Rates in the Venular Microvasculature

**Fig 3:**
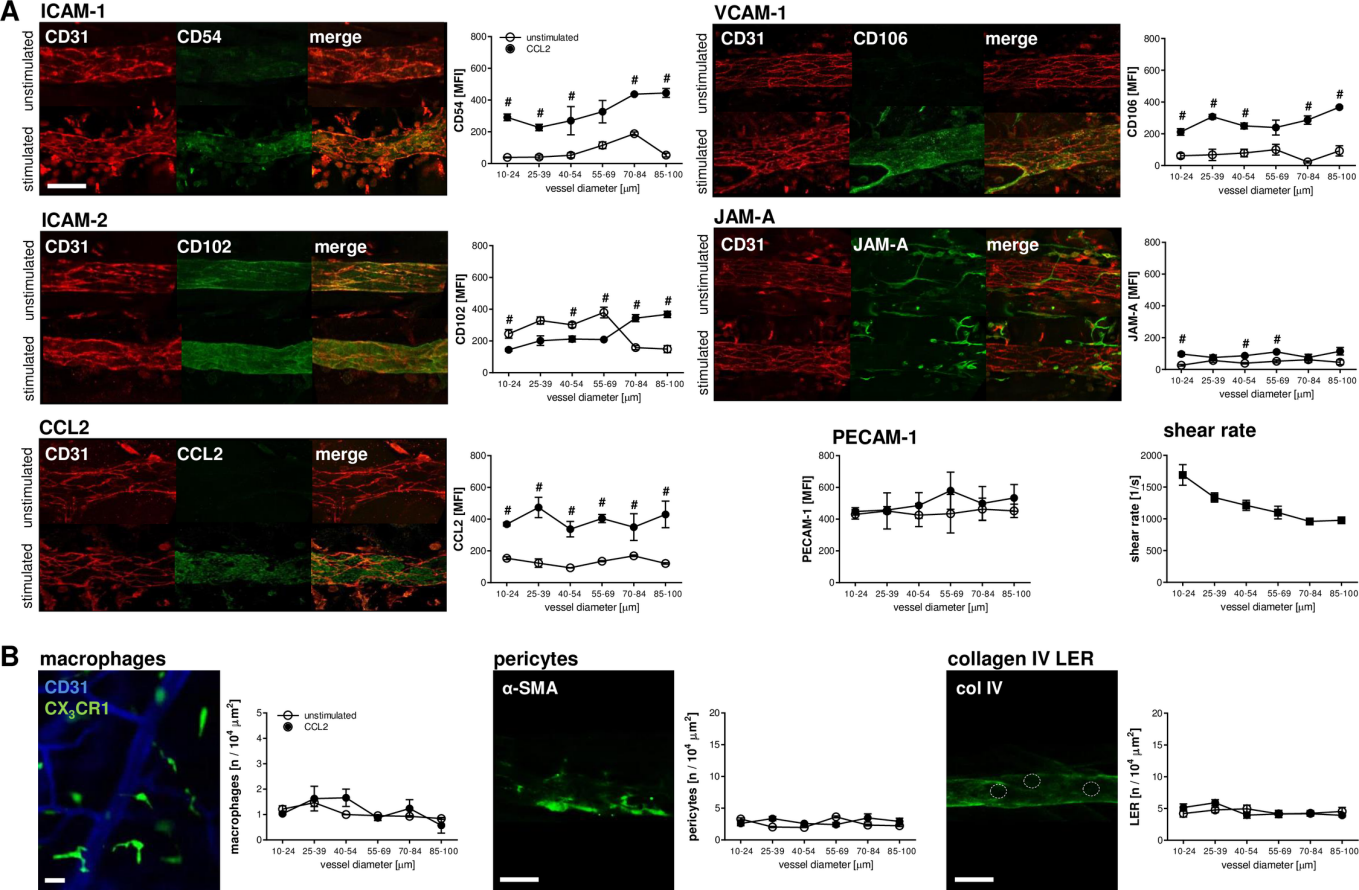
Endothelial expression of adhesion and signaling molecules, composition of the vascular wall, and shear rates in the venular microvasculature. Representative confocal microscopy images of ICAM-1/CD54, VCAM-1/CD106, ICAM-2/CD102, PECAM-1/CD31, JAM-A, and CCL2 expression in venular endothelial cells of the cremaster muscle of WT mice before (open dots) and after (filled dots) stimulation with CCL2 (**A**; scale bar 50 μm). Panels show quantitative expression levels of these proteins in dependency of the venular diameter as well as the corresponding venular shear rates (mean ± SEM; *n* = 3–4 per group; #*p* < 0.05 versus PBS). Panels (**B**) show representative images and quantitative data for the number of perivascular macrophages, pericytes, and collagen IV LERs of venular microvessels in dependency of the venular diameter (scale bar 50 μm; mean ± SEM; *n* = 3 per group).

To elaborate the molecular basis of this specific interaction profile of platelets, myeloid leukocytes, and endothelial cells in the CCL2-stimulated microvasculature, we analyzed the expression of key adhesion and signaling molecules on the luminal surface of venular microvessels by using confocal microscopy. In unstimulated control animals receiving an intrascrotal injection of phosphate-buffered saline (PBS), ICAM-1/CD54, ICAM-2/CD102, VCAM-1/CD106, JAM-A, and PECAM-1/CD31 as well as the C-C motif chemokine CCL2 were found to be nearly equally distributed throughout the entire venular microvasculature of the cremaster muscle. Whereas the expression of ICAM-1/CD54, VCAM-1/CD106, and CCL2 was significantly increased upon onset of CCL2-elicited inflammation, surface expression of ICAM-2/CD102, JAM-A, and PECAM-1/CD31 was not significantly altered (**Fig 3A**). Furthermore, the number of pericytes and perivascular macrophages as well as the number of collagen IV low expression regions (LERs) in the perivenular basement membrane (through which leukocytes pass this section of the venular wall [27–29]; **S5 Fig**) was not dependent on the vessel size (**Fig 3B**). In contrast, wall shear rates decreased in postcapillary vessel segments with increasing inner vessel diameters and reached the highest values in small-caliber venules (**Fig 3A**).

**Fig 4:**
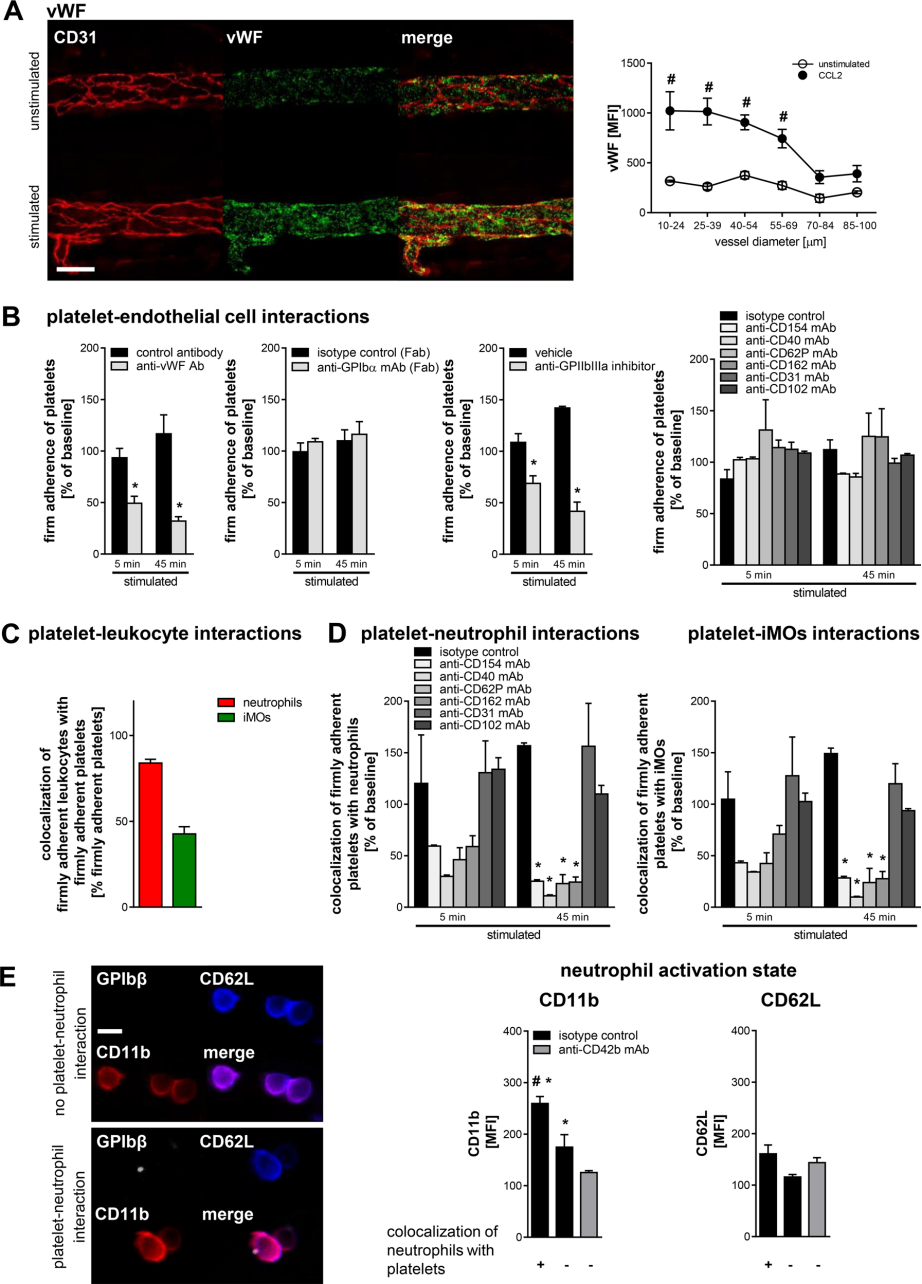
Mechanisms underlying interactions of platelets with endothelial cells, neutrophils, and iMOs. Representative confocal microscopy images of vWF expression in venular endothelial cells of the cremaster muscle before and after stimulation with CCL2 (**A;** scale bar: 50 μm). Panel shows quantitative expression levels of this protein in dependency of the venular diameter (mean ± SEM; *n* = 3 per group; #*p* < 0.05 versus PBS). Panels (**B**) show quantitative data for intravascular firm adherence of platelets to endothelial cells in CCL2-stimulated cremasteric venules of animals receiving blocking antibodies or inhibitors directed against vWF, GPIbα, GPIIbIIIa, CD40L/CD154, CD40, P-selectin/CD62P, PSGL-1/CD162, PECAM-1/CD31, or ICAM-2/CD102, or control antibodies or vehicle as assessed by multichannel in vivo microscopy on the cremaster muscle of CX_3_CR1^GFP/+^ mice (mean ± SEM; *n* = 4 per group; *p < 0.05 versus control antibody or isotype control or vehicle). Panels (**C, D**) show quantitative data for interactions (>30 s) between intravascularly adherent platelets and adherent neutrophils or iMOs in animals receiving blocking antibodies directed against CD40L/CD154, CD40, P-selectin/CD62P, PSGL-1/CD162, PECAM-1/CD31, or ICAM-2/CD102, or isotype control antibodies (mean ± SEM; *n* = 4 per group; *p < 0.05 versus isotype control). Representative multichannel in vivo microscopy images of Mac-1/CD11b and L-selectin/CD62L expression on the surface of intravascularly adherent neutrophils in CCL2-stimulated cremasteric venular microvessels (scale bar: 10 μm; **E**). Panels show quantitative data for the expression of Mac-1/CD11b or L-selectin/CD62L with respect to the relative localization of intravascularly adherent neutrophils to adherent platelets or in platelet-depleted animals (mean ± SEM; *n* = 4 per group; #*p* < 0.05 versus platelet-depleted; *p < 0.05 versus no colocalization with platelets).

Moreover, von Willebrand factor (vWF; which serves as an endothelial interaction partner of different platelet receptors, e.g., GPIIbIIIa) was predominantly expressed in endothelial junctions of small-caliber venules with diameters of less than 40 μm. Induction of inflammation significantly increased the expression of vWF in these segments of the venular microvasculature (**Fig 4A**).

### Mechanisms and Consequences of Interactions between Platelets, Myeloid Leukocytes, and Endothelial Cells in the Inflamed Microvasculature

In a next step, the functional relevance of these adhesion/signaling molecules and their interaction partners on platelets for endothelial cell interactions of platelets in small-caliber segments of the CCL2-stimulated cremasteric microvasculature was analyzed by multichannel in vivo microscopy. To avoid distraction by cumulative effects arising from a priori blockade of a defined protein in the course of the inflammatory response, blocking monoclonal antibodies directed against the different target proteins were administered after (and not prior to) the onset of inflammation and in vivo microscopy in the inflamed microvasculature was immediately performed. Whereas blockade of vWF or of its interaction partner GPIIbIIIa significantly reduced the number of intravascularly adherent platelets, inhibition of CD40, CD40L/CD154, GPIbα, P-selectin/CD62P, PSGL-1/CD162, ICAM-2/CD102, or PECAM-1/CD31 did not significantly alter platelet adherence to the inflamed endothelium (**Fig 4B**).

Interestingly, more than 85% of adherent neutrophils and the majority of adherent iMOs were observed to colocalize with firmly adherent platelets (**Fig 4C**). Blockade of CD40, P-selectin/CD62P, or their interaction partners CD40L/CD154 and PSGL-1/CD162 (but not of ICAM-2/CD102 or PECAM-1/CD31) significantly diminished the frequency of stable interactions between intravascularly adherent platelets and neutrophils or iMOs (**Fig 4D**).

To assess the role of these molecules for direct interactions between activated platelets and neutrophils or iMOs, additional in vitro experiments were performed. Platelet activation by adenosine diphosphate (ADP) significantly enhanced the number of neutrophils or iMOs binding to platelets. This increase was significantly reduced upon blockade of CD40 or its ligand CD40L/CD154 (but not upon blockade of P-selectin/CD62P; **S6 Fig**).

To further evaluate the effect of interactions between adherent platelets and neutrophils on the activation state of these immune cells, the expression of Mac-1/CD11b and L-selectin/CD62L (which represent commonly used leukocyte activation markers) was analyzed on adherent neutrophils in the inflamed microvasculature of the cremaster muscle by multichannel in vivo microscopy. Whereas the expression of Mac-1/CD11b was significantly higher on neutrophils adhering to intravascularly arrested platelets as compared to neutrophils adhering to the inflamed endothelium independently of platelets or adhering to the inner vessel wall in platelet-depleted animals, the expression levels of L-selectin/CD62L on neutrophils did not vary with their relative localization to platelets (**Fig 4E**).

Noteworthy, surface expression of P-selectin/CD62P (which is expressed by platelets and endothelial cells) did not differ between endothelial cells, endothelially adherent platelets, and endothelially adherent platelets capturing neutrophils or iMOs (**S7 Fig**).

### Effect of CD40L/CD154, P-selectin/CD62P, L-selectin/CD62L, or PSGL-1/CD162 on Activation of Leukocyte Integrins

**Fig 5:**
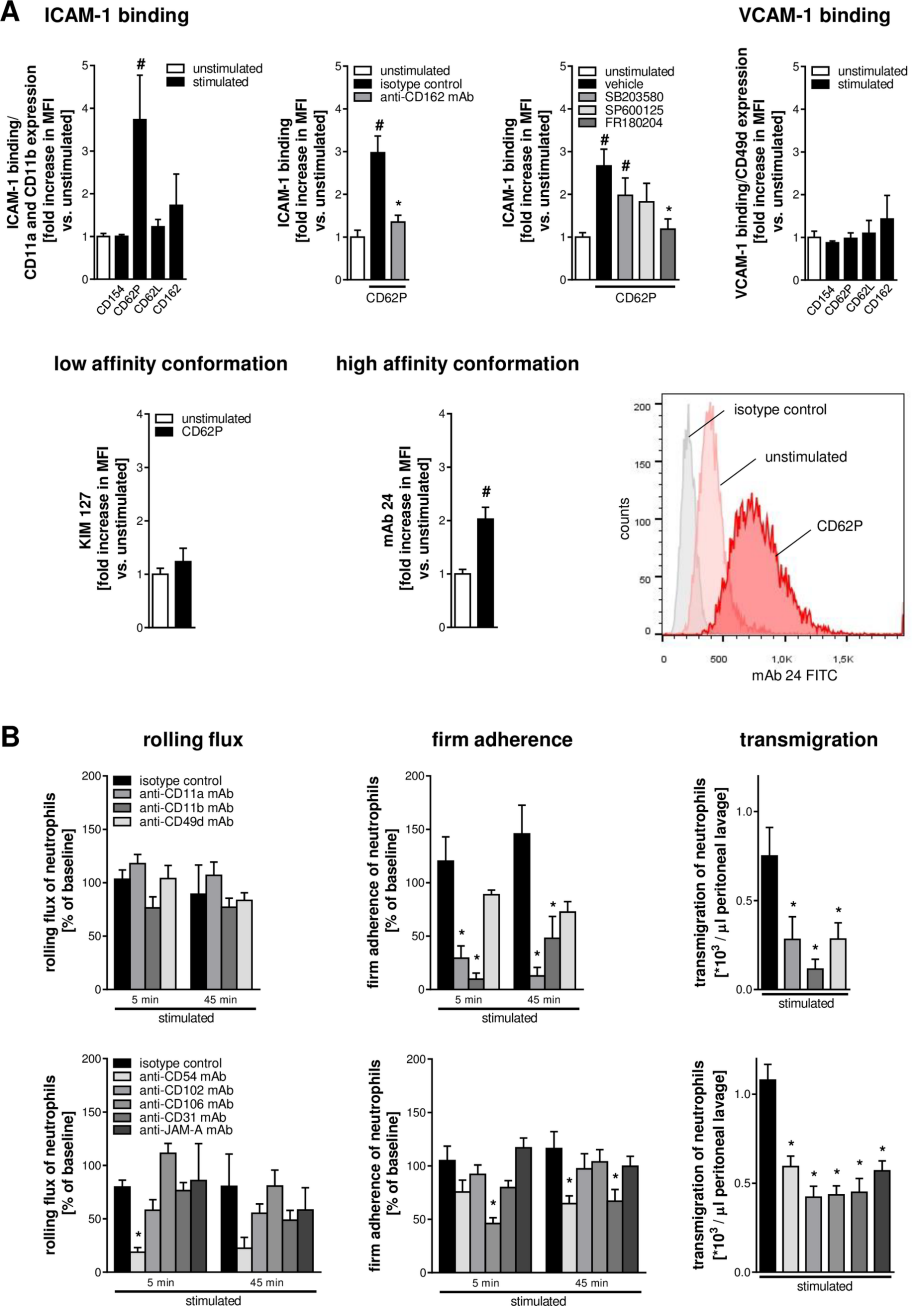
Consequences of interactions of neutrophils with platelets for the extravasation of neutrophils. Binding of ICAM-1/CD54 or VCAM-1/CD106 to neutrophils isolated from the peripheral blood of WT mice was assessed upon exposure to recombinant murine CD40L/CD154, P-selectin/CD62P, L-selectin/CD62L, or PSGL-1/CD162 by flow cytometry. P-selectin-elicited binding of ICAM-1/CD54 to murine neutrophils was quantified upon blockade of PSGL-1/CD162 or inhibition of MAPK (**A**, upper panels; mean ± SEM; *n* = 4–6 per group; #*p* < 0.05 versus unstimulated; *p < 0.05 versus isotype control or vehicle). Conformational changes of β2 integrins in human neutrophils were analyzed by using conformation-specific mAbs (**A**, lower panels). Using multichannel in vivo microscopy on the CCL2-stimulated cremaster muscle of CX_3_CR1^GFP/+^ mice, intravascular rolling flux and firm adherence of neutrophils were analyzed (mean ± SEM; *n* = 4 per group; #*p* < 0.05 versus unstimulated; *p < 0.05 versus isotype control or vehicle). Neutrophil extravasation was evaluated by using a peritonitis assay. Panels (**B**) show quantitative data for CCL2-challenged animals receiving blocking mAbs directed against Mac-1/CD11b, LFA-1/CD11a, VLA-4/CD49d, ICAM-1/CD54, ICAM-2/CD102, VCAM-1/CD106, PECAM-1/CD31, or JAM-A, or isotype control antibodies (mean ± SEM; *n* = 4–6 per group; *p < 0.05 versus isotype control).

Firm adherence of leukocytes to the microvascular endothelium is facilitated by interactions between leukocyte integrins in higher affinity conformation and their endothelial binding partners of the immunoglobulin superfamily (e.g., ICAM-1/CD54, VCAM-1/CD106) [2–6]. To characterize the consequences of interactions between platelets and neutrophils or iMOs on conformational changes of integrins on the surface of neutrophils and iMOs, the effect of recombinant P-selectin/CD62P, CD40L/CD154, L-selectin/CD62L, and PSGL-1/CD162 (which mediate this cellular interplay; see above) on leukocyte integrin affinity was analyzed. As a measure of conformational changes of leukocyte integrins, the binding capacity of these proteins for their interaction partners ICAM-1/CD54 (for β2 integrins) or VCAM-1/CD106 (for β1 integrins) was evaluated by flow cytometry. Static exposure to recombinant murine P-selectin/CD62P (but not to recombinant murine CD40L/CD154, L-selectin/CD62L, or PSGL-1/CD162) significantly enhanced the binding of neutrophils to ICAM-1/CD54 without altering the surface expression levels of β2 integrins. This effect was completely abolished upon blockade of PSGL-1/CD162 or upon inhibition of ERK1/2 MAPK (by compound FR180204), but not of p38 MAPK (by compound SB203580) or JNK (by compound SP600125) MAPK. In contrast, expression of the β1 integrin VLA-4/CD49d on the surface of neutrophils or binding of neutrophils to VCAM-1/CD106 was not significantly altered upon exposure to recombinant murine CD40L/CD154, P-selectin/CD62P, L-selectin/CD62L, or PSGL-1/CD162 (**Fig 5A**). In human neutrophils, exposure to recombinant human P-selectin induced the high-affinity conformation (but not the extended conformation) of β2 integrins, which allows binding to ICAM-1/CD54, as indicated by increased binding of the conformation-specific antibody “mAb 24” (**Fig 5A**).

Using in vivo microscopy on the cremaster muscle of CX_3_CR1^GFP/+^ mice, intravascular firm adherence of neutrophils in small-caliber venules was found to be significantly reduced upon blockade of the integrins LFA-1/CD11a or Mac-1/CD11b as well as upon inhibition of their interaction partner ICAM-1/CD54 or of PECAM-1/CD31. Furthermore, blockade of Mac-1/CD11b and—to a lesser extent—of LFA-1/CD11a or VLA-4/CD49d as well as of ICAM-1/CD54, ICAM-2/CD102, VCAM-1/CD106, PECAM-1/CD31, or JAM-A significantly decreased the extravasation of neutrophils to the inflamed peritoneal cavity (**Fig 5B**).

**Fig 6:**
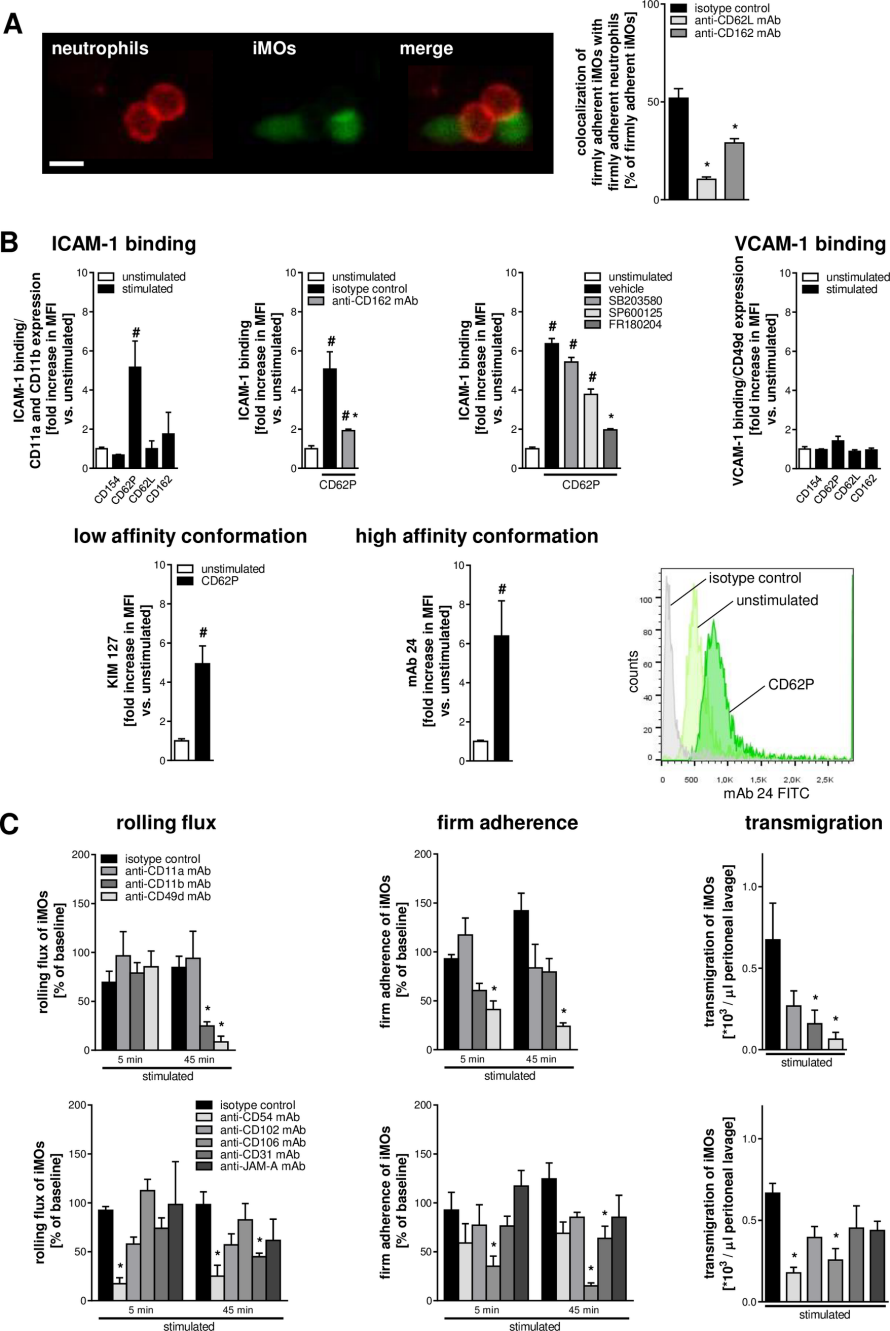
Consequences of interactions of iMOs with platelets and neutrophils for the extravasation of iMOs. Interactions of intravascularly adherent neutrophils and iMOs were analyzed by multichannel in vivo microscopy. Panel shows quantitative data for interactions (>30 s) between intravascularly adherent neutrophils and iMOs in animals receiving blocking mAbs directed against CD62L or PSGL-1/CD162, or istoype control antibodies (**A**; mean ± SEM; *n* = 4 per group; *p < 0.05 versus isotype control; scale bar 10 μm). Binding of ICAM-1/CD54 or VCAM-1/CD106 to iMOs isolated from the peripheral blood of WT mice was assessed upon exposure to recombinant murine CD40L/CD154, P-selectin/CD62P, L-selectin/CD62L, or PSGL-1/CD162 by flow cytometry (**B**; mean ± SEM; *n* = 4–6 per group; #*p* < 0.05 versus unstimulated; *p < 0.05 versus isotype control or vehicle). P-selectin-elicited binding of ICAM-1/CD54 to murine iMOs was quantified upon blockade of PSGL-1/CD162 or inhibition of MAPK (**B**, upper panels). Conformational changes of β2 integrins in human iMOs were analyzed by using conformation-specific mAbs (mean ± SEM; *n* = 4 per group; #*p* < 0.05 versus unstimulated; **B,** lower panels). Using multichannel in vivo microscopy on the CCL2-stimulated cremaster muscle of CX_3_CR1^GFP/+^ mice, intravascular rolling flux and firm adherence of iMOs were analyzed. Extravasation of iMOs was evaluated by using a peritonitis assay. Panels (**C**) show quantitative data for CCL2-challenged animals receiving blocking mAbs directed against Mac-1/CD11b, LFA-1/CD11a, VLA-4/CD49d, ICAM-1/CD54, ICAM-2/CD102, VCAM-1/CD106, PECAM-1/CD31, or JAM-A, or isotype control antibodies (mean ± SEM; *n* = 4–6 per group; *p < 0.05 versus isotype control).

In addition to platelets, the majority of intravascularly adherent neutrophils were found to colocalize with adherent iMOs. These neutrophil–monocyte interactions were dependent on L-selectin/CD62L and its ligand PSGL-1/CD162 (**Fig 6A**). Exposure to recombinant murine P-selectin/CD62P but not to recombinant murine CD40L/CD154, L-selectin/CD62L, or PSGL-1/CD162 significantly increased the binding capacity of iMOs for ICAM-1/CD54, whereas the binding capacity for VCAM-1/CD106 was only marginally altered. Similar to neutrophils, P-selectin-elicited ICAM-1/CD54 binding of iMOs was almost completely abolished upon blockade of PSGL-1/CD162 or upon inhibition of ERK1/2 MAPK (but not of p38 MAPK or JNK MAPK). Moreover, exposure to recombinant human P-selectin/CD62P induced the extended and the high-affinity conformation of β2 integrins on the surface of human neutrophils as indicated by increased binding of the conformation-specific antibodies “kim127” (extended conformation) and “mAb 24” (high-affinity conformation; **Fig 6B**).

As observed by in vivo microscopy on the cremaster muscle of CX_3_CR1^GFP/+^ mice, blockade of the β2 integrin Mac-1/CD11b or its ligand ICAM-1/CD54 significantly reduced the rolling flux of iMOs, whereas intravascular firm adherence of these inflammatory cells was significantly diminished upon blockade of the β1 integrin VLA-4/CD49d or its interaction partner VCAM-1/CD106 (**Fig 6C**). Extravasation of iMOs to the inflamed peritoneal cavity was significantly attenuated upon blockade of Mac-1/CD11b or VLA-4/CD49d or their interaction partners ICAM-1/CD54 or VCAM-1/CD106 (but not of ICAM-2/CD102, PECAM-1/CD31, or JAM-A).

**Fig 7:**
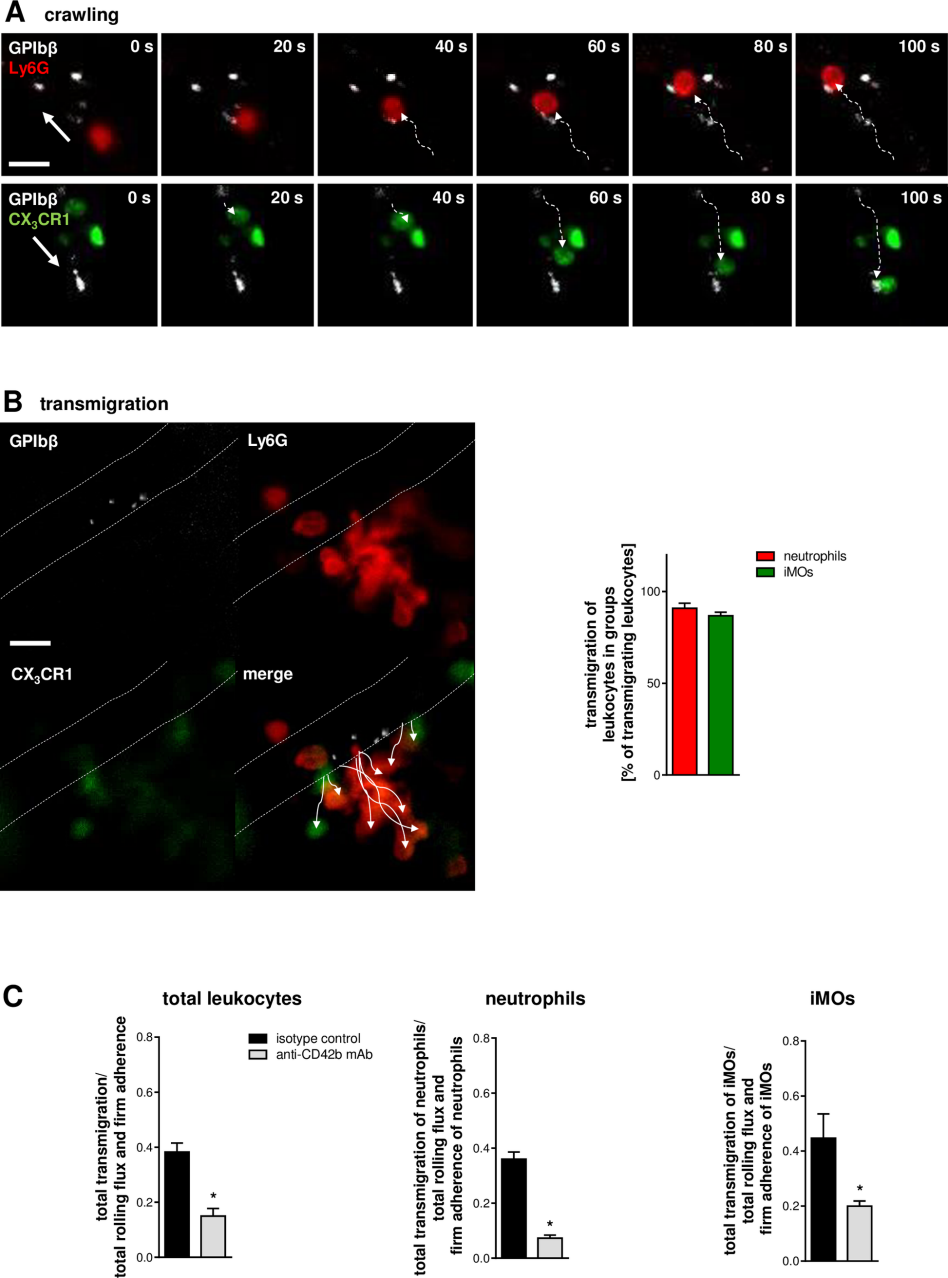
Role of platelets for the transmigration efficacy of leukocytes. Representative multichannel in vivo microscopy serial images of intravascularly adherent platelets (white) capturing an intraluminally crawling neutrophil (red) or iMO (green; **A**; arrows indicate direction of blood flow; scale bar 20 μm). Representative multichannel in vivo microscopy images as well as quantitative analysis of neutrophils and iMOs during their successive extravasation from sites of intravascularly adherent platelets (white; **B**; mean ± SEM; *n* = 4 per group; scale bar 20 μm). Panels (**C**) show quantitative data on the transmigration efficacy of total leukocytes, neutrophils, and iMOs in CCL2-stimulated cremasteric venules of animals receiving platelet-depleting mAbs or isotype control antibodies (mean ± SEM; *n* = 4 per group; *p < 0.05 versus isotype control).

To further characterize the nature of platelet-directed leukocyte extravasation, real-time and time-lapse records of multichannel in vivo microscopy experiments in the cremaster muscle of CX_3_CR1^GFP/+^ mice were analyzed. In these experiments, intravascularly adherent platelets were found to predominantly interact with neutrophils and iMOs by capturing these immune cells while rolling or crawling in the inflamed microvasculature (**Fig 7A; S2 Video**). Occasionally, rolling neutrophils (~20%) and iMOs (~10%) were observed to “take up” adherent platelets (**S3 Video**). Subsequently, neutrophils and iMOs arrested at sites of intravascularly adherent platelets and extravasated from these “hot spots” in the microvasculature to the inflamed tissue in a successive manner: more than 90% of neutrophils and about 80% of iMOs were found to transmigrate in “groups” (**Fig 7B**; **S4 Video**).

### Effect of Platelets on Leukocyte Extravasation Efficacy

In order to evaluate the effect of platelets on the overall extravasation efficacy of leukocytes in the venular microvasculature, the ratio of transmigrated leukocytes and intravascularly accumulated (rolling flux and firm adherence) leukocytes was determined in all venular vessel segments. Antibody-mediated platelet depletion significantly decreased the extravasation efficacy of total leukocytes, neutrophils, or iMOs (**Fig 7C**).

### Systemic Leukocyte Counts and Microhemodynamic Parameters

To assure intergroup comparability, systemic leukocyte counts and microhemodynamic parameters including blood flow velocity, inner vessel diameter, and wall shear rate were determined in each experiment. No significant differences were detected among experimental groups studying the functional relevance of different adhesion and signaling molecules for interactions between platelets, neutrophils, iMOs, and endothelial cells (**S2 Table**).

## Discussion

The directed recruitment of leukocytes to the site of injury or infection is indispensable for effective immune responses [2–6]. Recently, we have demonstrated that the spatiotemporal expression dynamics of selectins initiate the sequential endothelial cell interactions of neutrophils and iMOs in the acute inflammatory response [22]. How these immune cells subsequently “find” their sites of extravasation remains largely unknown.

In order to address this principal question, we sought to identify the individual sites that are utilized by neutrophils and iMOs to extravasate into inflamed tissue. Employing in vivo microscopy techniques, we were able to show that both immune cell populations predominantly establish their endothelial cell interactions in vessel segments of postcapillary venules exhibiting an inner diameter of approximately 25 μm and continue their subsequent passage through the vascular wall from the same sites. Since the endothelial expression of key adhesion and signaling molecules required for leukocyte extravasation, the structural composition of the vascular wall, and the distribution of macrophages in the perivascular tissue were found to be largely homogenous throughout the entire venular microvasculature, particularly the high shear stress present in small-caliber venules might favor the extravasation of neutrophils and iMOs at these vessel sites. This assumption is in line with previous in vitro data predicting shear stress to be supportive for interactions of leukocytes with endothelial cells in the microvasculature [30,31].

In addition to shear stress, however, platelets have recently been reported to contribute to the recruitment of leukocytes [10,11,13–16]. Here, we demonstrate that upon onset of inflammation, platelets are the first cellular blood components adhering to the endothelial surface of postcapillary venules, immediately before neutrophils and iMOs sequentially arrest in the same vascular regions. Interestingly, adherent platelets were almost exclusively detected at junctions of adjacent endothelial cells, which represent the sites from where neutrophils and iMOs initiate their transmigration process. With regard to these observations, we hypothesized that adherent platelets serve as “sentinels” defining the exit points of leukocytes in the microvasculature. In our experiments, we found that more than 85% of intravascularly adherent neutrophils and the majority of adherent iMOs were directly associated with adherent platelets. Platelet depletion completely abolished the directed extravasation of neutrophils and iMOs from these confined sites in low-diameter microvessels. Similarly, depletion of neutrophils resulted in random endothelial cell interactions and transmigration events of iMOs scattered throughout the entire venular microvasculature. Hence, our findings uncover a previously unrecognized role of platelets as “pathfinders” guiding leukocytes to their microvascular exit points. Since these events were observed under various inflammatory conditions, platelet-directed guidance of leukocytes to their site of extravasation might represent a more general inflammatory phenomenon.

Towards a more comprehensive, mechanistic understanding of this process, we systematically screened a variety of candidate adhesion molecules [32–42] for their functional relevance in mediating interactions of platelets with endothelial cells and neutrophils in this particular context. We were able to show that vWF, whose expression is pronounced in endothelial junctions of activated small-caliber venules, and GPIIbIIIa (but not GPIbα), which represent interaction partners of this protein on the surface of platelets, were indispensable for intravascular adherence of platelets at these sites. These data confirm recent observations as the early recruitment of platelets to inflamed liver sinusoids occurred via GPIIbIIa, but independently of GPIbα [12]. Moreover, we found that interactions of platelets and neutrophils strictly require CD40, P-selectin/CD62P, and their ligands CD40L/CD154 or PSGL-1/CD162, of which only CD40 and CD40L/CD154 were directly involved in mediating firm adherence of neutrophils to platelets. Our results extend recent reports describing a role of CD40 for interactions between platelets, endothelial cells, and leukocytes in atherosclerosis [43]. In this context, it is interesting that P-selectin/CD62P and PSGL-1/CD162 initiate signaling events in leukocytes [44–46]. Furthermore, circulating platelets have recently been observed to be capable of activating leukocytes (in 20%–30% of leukocyte–platelet interactions) [11]. Consequently, intravascularly adherent platelets might not only determine the microvascular exit points of neutrophils but might also actively promote the extravasation process of these immune cells at these sites. Confirming this hypothesis, we found that neutrophils recruited to intravascularly adherent platelets exhibit a higher activation state as compared to neutrophils arresting on the microvascular endothelium in the absence of platelets. Specifically, ligation of PSGL-1/CD162 by P-selectin/CD62P under these static conditions induced the high-affinity conformation of β2 integrins on the surface of neutrophils (as opposed to the induction of the extended conformation of β2 integrins in rolling leukocytes [44,47,48]) via ERK1/2 MAPK-dependent signaling events. Moreover, neutrophil extravasation at the sites of adherent platelets was almost completely inhibited upon blockade of the integrins LFA-1/CD11a, Mac-1/CD11b, and VLA-4/CD49d or ICAM-1/CD54, ICAM-2/CD102, PECAM-1/CD31, VCAM-1/CD106, and JAM-A. In summary, our data indicate that intravascularly adherent platelets capture neutrophils through CD40 and CD40L/CD154. These events, in turn, enable P-selectin/CD62P to induce the high-affinity conformation in neutrophil β2 integrins via PSGL-1/CD162 and ERK1/2 MAPK thereby promoting the passage of these immune cells through the vessel wall.

Secretion products of emigrating neutrophils have been documented to facilitate the sequential recruitment of iMOs under prolonged or chronic inflammatory conditions [1]. In the initial inflammatory response, however, we found that intravascularly adherent platelets and neutrophils cooperatively recruit iMOs through CD40-, CD40L/CD154-, P-selectin/CD62P-, L-selectin/CD62L-, and PSGL-1/CD162-dependent interactions, of which only CD40 and CD40L/CD154 directly promoted adherence of iMOs to platelets. Ligation of PSGL-1/CD162 by P-selectin/CD62P, in contrast, led to the induction of the extended and high-affinity conformation of β2 integrins on the surface of iMOs via induction of ERK1/2 MAPK-dependent signaling events. Monocyte LFA-1/CD11a, Mac-1/CD11b, and VLA-4/CD49d together with their endothelial interaction partners ICAM-1/CD54 and VCAM-1/CD106 subsequently consolidated firm adherence of iMOs at the sites of intravascularly adherent platelets and neutrophils and promoted the extravasation of these immune cells to the inflamed tissue.

Having deciphered the mechanisms underlying platelet-directed leukocyte recruitment to their sites of extravasation, we sought to characterize the biological relevance of this process. It is well documented that platelets are critically involved in the pathogenesis of acute inflammatory conditions such as ischemia-, reperfusion-, transfusion-, thermally, or acid-induced injury and sepsis [11,15,16,49–52] as well as of chronic inflammatory pathologies including autoimmune disorders [53] or atherosclerosis [54]. With regard to these reports and our present findings, we proposed that platelet-directed leukocyte trafficking enhances the efficacy of immune cell recruitment to the site of inflammation. The breaching of the venular basement membrane is considered as the “rate-limiting” step in leukocyte extravasation, which has to be accomplished by the first leukocytes encountering this vascular structure [55]. Accordingly, we found that in the absence of platelets, the overall recruitment of leukocytes to the inflamed tissue is severely compromised, as myeloid leukocytes interacted with the venular wall at multiple sites in the microvasculature. In contrast, platelet-directed extravasation of neutrophils and iMOs promoted successive, uninterrupted extravasation of these immune cells in “groups” by focusing leukocyte diapedesis to few, confined microvascular spots. Thus, these data clearly demonstrate that platelet-directed guidance of leukocytes to distinct sites of extravasation increases the efficacy of the leukocyte recruitment process thereby enabling effective immune responses. To what extent platelets that only transiently interact with endothelial cells (e.g., intravascularly rolling platelets) or with adherent leukocytes as well as platelets taken up by rolling leukocytes contribute to leukocyte extravasation, however, cannot clearly be stated.

**Fig 8:**
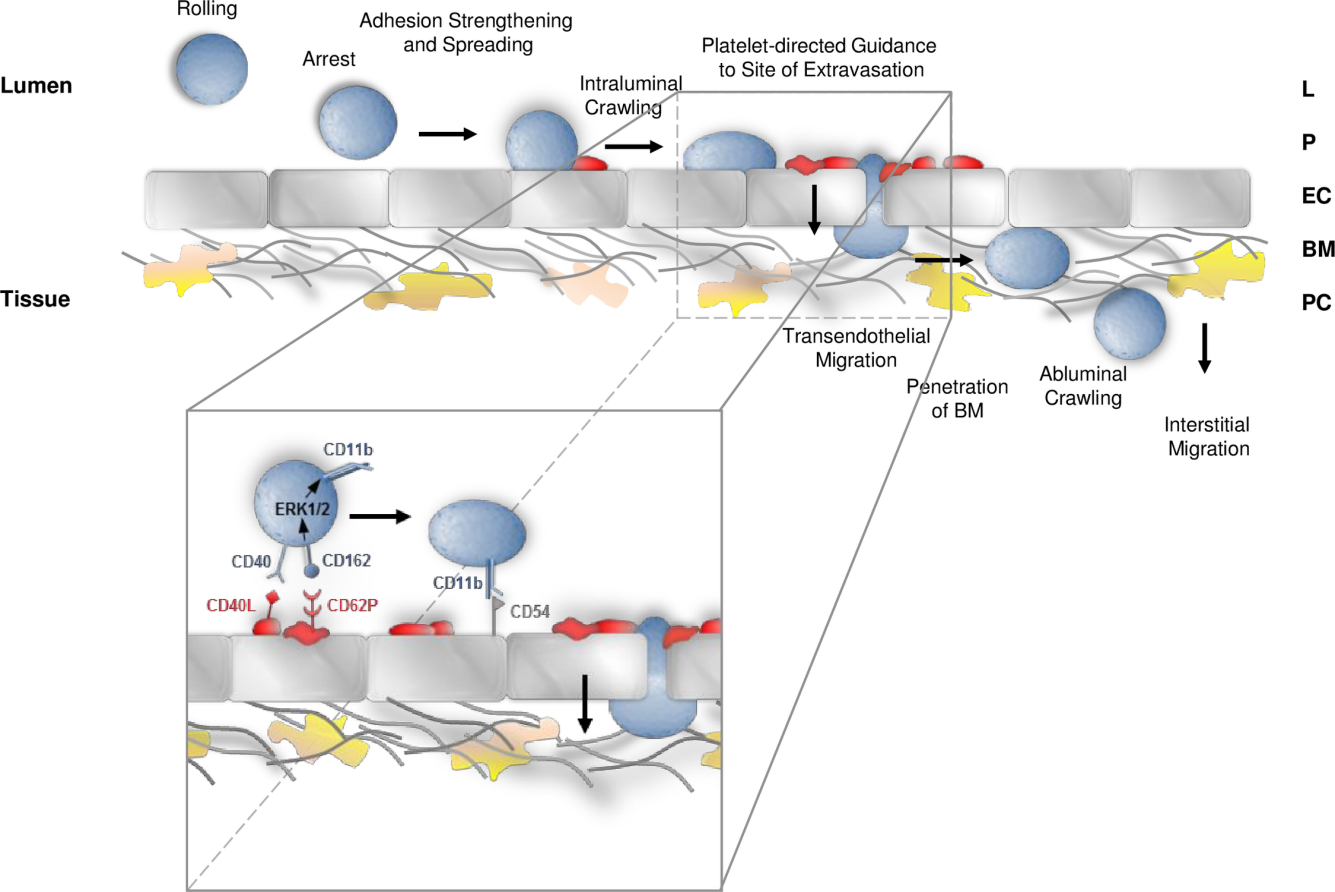
Graphical synopsis. Schematic illustration of the sequential steps in the leukocyte recruitment cascade including platelet-directed guidance of leukocytes to their site of extravasation.

In conclusion, our experimental data assign a previously unrecognized role to platelets as “pathfinders” guiding leukocytes to their exit points in the inflamed microvasculature: upon onset of inflammation, platelets immediately arrest at distinct sites in venular microvessels enabling these cellular blood components to capture neutrophils and, in turn, iMOs. The cellular crosstalk arising from these interactions leads to an activation of surface-expressed leukocyte integrins, which subsequently promotes the successive extravasation of neutrophils and iMOs from these “hot spots” to the perivascular tissue. Blockade of this cellular partnership results in misguided, inefficient leukocyte responses collectively uncovering a platelet-directed, spatiotemporally organized, multicellular crosstalk that is essential for effective trafficking of leukocytes to their sites of inflammation. These findings define platelet-directed guidance of leukocytes to confined sites of extravasation as a critical step in the recruitment process of immune cells (**Fig 8**), which might emerge as a promising therapeutic target for the prevention and treatment of inflammatory pathologies.

## Material and Methods

### Ethics Statement

According to the guidelines of the local governmental authorities (“Regierung von Oberbayern”), animal welfare was ensured. All animal experiments were approved by the local governmental authorities (“Regierung von Oberbayern”). For all surgical procedures, animals were anesthetized with ketamine and xylazine (see below).

### Animals

Male C57BL/6 mice were purchased from Charles River (Sulzfeld, Germany). Male CX_3_CR1^GFP/+^ mice were generated as described previously and backcrossed to the C57BL/6 background for six to ten generations [56]. All experiments were performed using mice at the age of 10 to 12 wk. Animals were housed under conventional conditions with free access to food and water. The experiments were performed according to German legislation for the protection of animals and approved by the local government authorities.

### Reagents

Recombinant murine CCL2 (0.3 μg in 400 μl PBS i.s.; R&D Systems, Nordenstadt, Germany), TNF (0.5 μg in 400 μl PBS intrascrotally (i.s.); R&D Systems), IL-1β (0.05 μg in 400 μl PBS intrascrotally (i.s.); R&D Systems), or IFN-γ (1.0 μg in 400 μl PBS intrascrotally (i.s.); R&D Systems) was used to induce expression of endothelial adhesion/signaling molecules and/or (subsequent) leukocyte recruitment. An anti-Ly-6G monoclonal antibody (mAb; clone 1A8; 150 μg intravenously (i.v.); 24 h and 6 h prior to induction of inflammation; BD Biosciences, San Jose, CA, US) was used for the depletion of neutrophils. An anti-GPIbα (CD42b) mAb (clone Xia.B2; 50 μg i.v.; 24 h and 6 h prior to induction of inflammation; emfret Analytics, Eibelstadt, Germany) was used for the depletion of platelets. Platelet–endothelium and platelet–leukocyte interactions were analyzed upon administration of blocking Abs and inhibitors: anti-vWF polyclonal Ab (100 μg in 100 μl PBS intraarterially (i.a.); Dako, Dako Deutschland GmbH, Hamburg, Germany), anti-GPIbα Fab-fragment (clone Xia.B2; 60 μg in 100 μl PBS i.a.; emfret Analytics), GR 144053 (GPIIbIIIa antagonist; 0.25 mg in 100 μl PBS i.a; Tocris Bioscience, Bristol, UK), anti-CD154 mAb (clone MR1; 50 μg in 100 μl PBS i. a.; eBiosciences), anti-CD40 mAb (clone 1C10; 50 μg in 100 μl PBS i.a.; eBiosciences), anti-CD62P mAb (clone RB40.34; 50 μg in 100 μl PBS i.a.; BD Biosciences), anti-CD162 mAb (clone 4RA10; 50 μg in 100 μl PBS i.a. or i.v.; BD Biosciences), or anti-CD31 mAb (clone MEC 13.3; 50 μg in 100 μl PBS i.a.; BD Biosciences). Leukocyte responses were analyzed upon administration of blocking mAbs: anti-CD11a mAbs (clone M17/4; 50 μg in 100 μl PBS i.a.; Biolegend, San Diego, CA, US), anti-CD11b mAb (clone M1/70; 50 μg in 100 μl PBS i.a.; Biolegend), anti-CD49d mAb (clone R1-2; 50 μg in 100 μl PBS i.a.; Biolegend), anti-CD54 mAb (clone YN1/1.7.4; 50 μg in 100 μl PBS i.a.; Biolegend), anti-CD102 mAb (clone 3C4; 50 μg in 100 μl PBS i.a.; Biolegend), anti-CD106 mAb (clone 425; 50 μg in 100 μl PBS i.a.; Biolegend), anti-CD31 mAb (clone MEC 13.3; 50 μg in 100 μl PBS i.a.; BD Biosciences) or anti-JAM-A mAb (clone BV-11; 50 μg in 100 μl PBS i.a.; Merck Millipore, Darmstadt, Germany). Classical/iMOs (and neutrophils) were labeled with anti-Ly6C PE mAb (clone HK1.4, 5 μg in 100 μl PBS i.a.; Biolegend), NK cells were labeled with an anti-NK1.1 PE mAb (clone PK136, 5 μg in 100 μl PBS i.a.; eBioscience), and T cells were labeled with an anti-CD3 PE mAb (clone 17A2, 5 μg in 100 μl PBS i.a.; Biolegend). Neutrophils were labeled with an anti-Ly6G PE mAb (clone 1A8; 5 μg in 100 μl PBS i.a.; BD Biosciences), platelets were visualized by a rat IgG derivative against the GPIbβ subunit of the murine platelet/megakaryocyte-specific GPIb-V-IX complex (X649; DyLight 649-labeled, noncytotoxic and not interfering with platelet adhesion and aggregation in vitro and in vivo; 3 μg in 100 μl PBS i.a.; emfret Analytics) [57,58]. Endothelial junctions were visualized with a nonblocking PE-labeled anti-CD31 mAb (clone 390; 3 μg in 100 μl PBS i.a; eBiosciences).

### In Vivo Microscopy on the Cremaster Muscle

#### Surgical procedure

The surgical preparation of the mouse cremaster muscle was performed as originally described by Baez with minor modifications [59–61]. Mice were anesthetized using a ketamine/xylazine mixture (100 mg/kg ketamine and 10 mg/kg xylazine), administrated by i.p. injection. The left femoral artery was cannulated in a retrograde manner for administration of microspheres and antibodies. The right cremaster muscle was exposed through a ventral incision of the scrotum. The muscle was opened ventrally in a relatively avascular zone, using careful electrocautery to stop any bleeding, and spread over the pedestal of a custom-made microscopy stage. Epididymis and testicle were detached from the cremaster muscle and placed into the abdominal cavity. Throughout the procedure as well as after surgical preparation during in vivo microscopy, the muscle was superfused with warm buffered saline.

#### Experimental protocols

In a first set of experiments, three postcapillary vessel segments with vessel diameters of 10–24 μm, 25–39 μm, 40–54 μm, 55–69 μm, 70–84 μm, or 85–100 μm were randomly chosen in a central area of the spread-out cremaster muscle among those that were at least 150 μm away from neighboring postcapillary venules and did not branch over a distance of at least 150 μm. In vivo microscopy measurements of leukocyte intravascular rolling and adherence and as well as transmigration were performed in CX_3_CR1^GFP/+^ mice 6 h after i.s. injection of PBS or i.s. stimulation with CCL2, TNF, IL-1β, or IFN-γ and administration of depleting or isotype control antibodies (*n* = 3–4 per group). In the same experiments, intravascular rolling and adherence of platelets were measured.

In a separate set of experiments, platelet and leukocyte responses were analyzed upon intrascrotal injection of PBS (“time point 0”) or 0.5 h, 1.0 h, 1.5 h, 2.0 h, and 6.0 h after i.s. injection of CCL2 or TNF (*n* = 3–4 each group).

To analyze the mechanisms underlying platelet–endothelial cell as well as platelet–neutrophil interactions, baseline in vivo microscopy measurements of neutrophil rolling and adherence were performed in three postcapillary vessel segments with a diameter of 20–35 μm 6 h after i.s. stimulation with CCL2. Subsequently, blocking Abs or inhibitors were applied and the in vivo microscopy measurements were repeated 5 and 45 min after the administration of mAbs/inhibitors (*n* = 4). In further experiments, baseline in vivo microscopy measurements of leukocyte rolling and adherence were performed in three postcapillary vessel segments with a diameter of 20–35 μm 6 h after i.s. stimulation with CCL2. Subsequently, blocking antibodies against integrins or members of the immunoglobulin superfamily were applied i.a., and measurements were repeated after 5 and 45 min in the same vessel segments. Additionally, the intraluminal crawling behavior of leukocytes was analyzed for 30 min in a single postcapillary venule 15 min after the administration of mAbs (*n* = 4).

After in vivo microscopy (~ 60 min after administration of mAbs), blood samples were collected by cardiac puncture for the determination of systemic leukocyte and platelet counts using a Coulter ACT Counter (Coulter Corp., Miami, Florida, US) or Procyte Dx (IDDEXX, Maine, US; for differential leukocyte counts). Anesthetized animals were then killed by bleeding to death. In selected experiments, nonblocking PE-labeled anti-Ly-6G mAbs (visualization of neutrophils), nonblocking PE-labeled anti-Ly-6C mAbs (visualization of GFP^low^ classical/iMOs in CX_3_CR1^GFP/+^ mice), nonblocking PE-labeled anti-NK1.1 mAbs (visualization of NK cells), nonblocking PE-labeled anti-CD3 mAbs (visualization of T cells), PE-labeled anti-CD31 mAbs (visualization of endothelial junctions), or DyLight 649-labelled immunoglobulin derivates against the GPIbβ-subunit of the murine platelet/megakaryocyte-specific GPIb-V-IX complex (visualization of platelets) were used.

#### Peritonitis assay

After 6 h of i.p. stimulation with CCL2, mice were sacrificed and their peritoneal cavity was washed with 10 ml of ice-cold PBS as described previously. The total number of leukocytes recovered from the peritoneal lavage fluid was analyzed by using a Coulter A C T counter (Coulter Corp.). Samples were then labeled with anti-CD45 APC-Cy7 mAb (clone 30-F11; BD Bioscience), anti-CD11b FITC mAb (clone M1/70; eBioscience, San Diego, CA, US), anti-GR-1 PE mAb (clone RB6-8C5; eBioscience), anti-CD115 APC mAb (clone AFS98; eBioscience), and anti-F4/80 eFluor450 mAb (clone BM8; eBioscience) for 30 minutes on ice. Erythrocytes were lysed with lysing solution (1:10; BD FACS lysing solution; BD Bioscience). After two washing steps, leukocytes were resuspended in 250 μl PBS.

Using flow cytometry (Gallios; Beckman Coulter Inc, Brea, CA, USA), myeloid leukocytes were detected by expression of CD45 and CD11b as well as by the absence of F4/80. Therefore, neutrophils were identified by high expression of Gr-1 and low expression of CD115, iMOs by high expression of Gr-1 and CD115, and resident monocytes by high expression of CD115 as well as by low expression of Gr-1.

#### In vivo microscopy

The setup for in vivo microscopy was centered around an AxioTech-Vario 100 Microscope (Zeiss MicroImaging GmbH, Goettingen, Germany), equipped with a Colibiri LED light source (Zeiss MicroImaging GmbH) for fluorescence epi-illumination microscopy as described previously [62]. Light was directed onto the specimen via filter set 62 HE (Zeiss MicroImaging GmbH) fitted with dichroic and emission filters [TFT 495 + 610 (HE); TBP 527 + LP615 (HE)]. Microscopy images were obtained with an AxioCam Hsm digital camera using a 20x water immersion lens (0.5 NA, Zeiss MicroImaging Gmbh). The images were processed with AxioVision 4.6 software (Zeiss MicroImaging GmbH).

#### Quantification of leukocyte kinetics and microhemodynamic parameters

In vivo microscopy records were analyzed offline using the imaging software ImageJ (National Institutes of Health, Bethesda, MD). GFP^neg^, GFP^high^, and GFP^low^ cells were distinguished by the analysis of their fluorescence intensities as described previously [22]. Rolling leukocytes were defined as those moving slower than the associated blood flow and quantified for 60 s per venule. Firmly adherent cells were determined as those resting in the associated blood flow for 30 s and related to the luminal surface per 100 μm vessel length. Concomitantly, colocalization (= direct contact) in the same focal plane of all firmly adherent platelets and firmly adherent leukocytes as well as colocalization (= direct contact) of leukocytes or platelets with endothelial junctions was assessed.

Centerline blood flow velocity was determined by measuring the distance between several images of one fluorescent bead under stroboscopic illumination. An empirical factor of 0.625 was used to convert centerline velocities to mean blood flow velocities [63]. Wall shear rates were estimated as 4.9 (8v_b_/d), v_b_ being mean blood flow velocity and d the diameter of the vessel [64,65].

### Experimental Groups

Animals were assigned randomly to the following groups: In a first set of experiments, CX_3_CR1^GFP/+^ mice received neutrophil or platelet depleting mAbs or isotype control antibodies prior to 360 min of i.s. stimulation with recombinant murine CCL2, TNF, IL-1β, IFN-γ, or injection of PBS (*n* = 3 per group). In further experiments, CX_3_CR1^GFP/+^ mice received an i.a. injection of anti-vWF Ab, anti-GPIbα Fab fragment, GR 144053, anti-CD40 mAb, anti-CD40L/CD154 mAb, anti-P-selectin/CD62P mAb, anti-PSGL-1/CD162 mAb, anti-PECAM-1/CD31 mAb, isotype control antibodies/Fab fragments/drug vehicle prior to 6 h of i.s. stimulation with recombinant murine CCL2 (*n* = 4 per group). In a next series of experiments, CX_3_CR1^GFP/+^ mice received an i.a. injection of blocking mAbs directed against LFA-1/CD11a, Mac-1/CD11b, VLA-4/CD49d, ICAM-1/CD54, ICAM-2/CD102, VCAM-1/CD106, PECAM-1/CD31, or JAM-A or isotype control antibodies after 6 h of i.s. stimulation with recombinant murine CCL2 (*n* = 5 per group). Finally, C57BL/6 mice received an i.v. injection of mAbs directed against LFA-1/CD11a, Mac-1/CD11b, VLA-4/CD49d, ICAM-1/CD54, ICAM-2/CD102, VCAM-1/CD106, PECAM-1/CD31, or JAM-A 6 h prior to intraperitoneal stimulation with CCL2 (*n* = 7 per group).

### Confocal Microscopy

For the analysis of PECAM-1, ICAM-1/CD54, ICAM-2/CD102, VCAM-1/CD102, JAM-A, CCL2, or vWF expression on endothelial cells of postcapillary venules, for the analysis of collagen IV expression in the venular basement membrane as well as for the analysis of the pericyte distribution in postcapillary venules, excised mouse cremaster muscles (6 h after intrascrotal injection of CCL2 or PBS) were fixed in 2% paraformaldehyde. Tissues were then blocked and permeabilized in PBS, supplemented with 10% goat serum (Sigma Aldrich) and 0.5% Triton X-100 (Sigma Aldrich). After incubation at 4°C for 12 h with antibodies directed against PECAM-1 (CD31 goat IgG; Santa Cruz Biotechnology, Dallas, Texas, US) and CD54 (rat anti-mouse; Biolegend), PECAM-1 (CD31 goat IgG; Santa Cruz Biotechnology) and CD102 (rat anti-mouse; Biolegend), PECAM-1 (CD31 goat IgG; Santa Cruz Biotechnology) and CD106 (rat anti-mouse; Biolegend), PECAM-1 (CD31 goat IgG; Santa Cruz Biotechnology) and JAM-A (rat anti-mouse; Merck Millipore), PECAM-1 (CD31 goat IgG; Santa Cruz Biotechnology) and vWF (rabbit anti-human; Dako), PECAM-1 (CD31 goat IgG; Santa Cruz Biotechnology) and CCL2 (rabbit polyclonal; abcam, Cambridge, UK), PECAM-1 (CD31 goat IgG; Santa Cruz Biotechnology) and α-SMA (rabbit antimouse; abcam), PECAM-1 (CD31 goat IgG; Santa Cruz Biotechnology) and collagen IV (rabbit anti-mouse; abcam) tissues were incubated for 180 min at room temperature with an Alexa Fluor 633-linked donkey antigoat antibody (molecular probes) and then with an Alexa Fluor 488-linked goat antirat antibody (molecular probes) or with an Alexa Fluor 633-linked donkey antigoat antibody (molecular probes) and an Alexa Fluor 488-linked chicken antirabbit antibody (molecular probes). Immunostained tissues were mounted in PermaFluor (Beckman Coulter, Fullerton, CA) on glass slides. Confocal z-stacks typically covering 30 μm (z-spacing 0.5 μm) were acquired using a Leica SP5 confocal laser-scanning microscope (Leica Microsystems, Wetzlar, Germany) with an oil-immersion lens (Leica; 63x; NA 1.40). The fluorescence signal of CD31, CD54, CD102, CD106, JAM-A, vWF, or CCL2 was only quantified on the surface of endothelial cells of postcapillary venules not measuring the fluorescence signal of these adhesion and signaling molecules on leukocytes and platelets (easily identified by expression of PECAM-1 and morphological characteristics), and the background signal was subtracted.

### Flow Cytometry

To analyze the effect of CD40L/CD154, P-selectin/CD62P, L-selectin/CD62L, or PSGL-1/CD162 on the expression profiles of LFA-1/CD11a, Mac-1/CD11b, or VLA-4/CD49d on murine neutrophils and monocytes, anticoagulated whole blood samples were incubated (30 min; 37°C) with recombinant mouse CD40L/CD154 (10 μg/ml; PeproTech, Rocky Hill, New Jersey, US), P-selectin/CD62P (0.01, 0.1, 1, or 10 μg/ml; R&D Systems), recombinant mouse L-selectin/CD62L (0.01, 0.1, 1, or 10 μg/ml; R&D Systems), recombinant mouse PSGL-1/CD162 (0.01, 0.1, 1, or 10 μg/ml; R&D Systems), or PBS as negative control. Additionally, the effect of CCL2 or PMA on the expression profiles of LFA-1/CD11a, Mac-1/CD11b, or VLA-4/CD49d on murine blood leukocytes was analyzed by incubation (30 min; 37°C) with CCL2 (100 ng/ml; R&D Systems), PMA (50 ng/ml; Sigma Aldrich, St. Louis, US), or PBS as negative control. After washing, cells were incubated with primary antibodies directed against CD45, CD11b, GR-1, CD115, CD62L, CD44, and CD162 on ice. Isotype-matched controls were used in all experiments. After lysis of erythrocytes, stained cells were analyzed on a flow cytometer (Gallios, Beckmann Coulter). Approximately 20,000 gated events were collected in each analysis.

For the analysis of integrin activation, murine peripheral blood cells were isolated from male C57BL/6 mice, anticoagulated, and suspended in Hanks balanced salt solution containing 1 mM CaCl2 and MgCl2 (Life Technologies, Carlsbad, US). Cells were exposed to PMA (50 ng/ml, Sigma-Aldrich), CCL2 (100 ng/ml, R&D Systems), or PBS as negative control, in the presence of ICAM-1/Fc (10 μg/ml, R&D Systems), VCAM-1/Fc (10μg/ml, R&D Systems), and PE-conjugated antihuman IgG1 (Fc-specific, Southern Biotechnology) for 5 min at 37°C. In further experiments, cells were treated with a blocking mAb against PSGL-1/CD162 or isotype control antibody, SB203580 (25 μM; Sigma Aldrich), SP600125 (5 μM; Sigma Aldrich), FR180204 (15 μM; Sigma Aldrich) or vehicle. After washing, cells were labeled with antibodies directed against CD45, CD11b, CD115, and Gr1. Binding of ICAM-1 or VCAM-1 was measured by a flow cytometer (Gallios, Beckmann Coulter). The results were analyzed with FlowJo Software (Treestar).

Moreover, binding of neutrophils or iMOs to platelets was measured by flow cytometry after incubation of murine peripheral blood with the potent platelet-activating substance ADP (10 μM; 10 min 37°C; Sigma Aldrich, St. Louis, Missouri, US) or PBS as well as with blocking monoclonal antibodies directed against CD62P, CD40L/CD154, PECAM-1/CD31, ICAM-2/CD102, or isotype control antibodies. Myeloid leukocyte subsets were identified by their expression of CD45, CD11b, GR-1, and CD115 (see above). Platelets were stained with a primary anti-GPIIb/IIIa antibody (clone JON/A; emfret Analytics, Eibelstadt, Germany). After lysis of erythrocytes, stained cells were analyzed on a flow cytometer (Gallios, Beckmann Coulter). Approximately 20,000 gated events were collected in each analysis. Binding of neutrophils or iMOs to ADP-activated platelets was measured by determining the number of JON/A-positive and -negative neutrophils or iMOs.

In a next set of experiments, anticoagulated whole human blood was obtained from healthy human donors, stimulated with recombinant human P-selectin/CD62P (10 μg/ml; R&D Systems) or PBS. Neutrophils, nonclassical (CD14^+^ CD16^++^), intermediate (CD14^++^ CD16^+^), and classical/iMOs (CD14^++^ CD16^-^) were distinguished by size and granularity as well as by their expression of CD45, CD16, and CD14. Binding of mAb24 (detection of high-affinity conformation of β2 integrins; mouse anti-human, monoclonal; abcam) or KIM127 (detection of extended conformation of β2 integrins; mouse antihuman, monoclonal; gift from M. Sperandio) was measured by flow cytometry.

### Statistics

Data analysis was performed with a statistical software package (SigmaStat for Windows; Jandel Scientific). Rank Sum test (two groups) or ANOVA-on-ranks followed by the Dunnett test (> two groups) were used for the estimation of stochastic probability in intergroup comparisons. Mean values and SEM are given. *p*-Values < .05 were considered significant.

## Supporting Information

S1 Data Excel file containing in separate sheets the numerical data for all figures. (XLSX)

S1 Fig In vivo microscopy analysis of different leukocyte subsets. Using multichannel in vivo microscopy on the cremaster muscle of CX_3_CR1^GFP/+^ mice, inflammatory/classical monocytes (GFP^low^ Ly6C^+^), and ncMOs (GFP^high^ Ly6C^-^) were differentiated by their relative fluorescence intensity of GFP and by in vivo immunostaining for Ly6C, GFP^+^ tissue macrophages were identified by their morphology. Representative images (**A**; scale bar: 30 μm) and quantitative data for the expression of Ly6C in intravascular or extravascular GFP^high^ and GFP^low^ leukocytes are shown. NK1.1^+^ NK cells and CD3^+^ T cells were identified by in vivo immunostaining for NK1.1 or CD3. Representative images (**B**; scale bar: 30 μm) and quantitative data for the expression of NK1.1 or CD3 in rolling and adherent total leukocytes or GFP^+^ leukocytes are shown (mean ± SEM for *n* = 4 per group).
(TIF)

S2 Fig Interactions of platelets, neutrophils, and iMOs in the TNF-stimulated microvasculature. Interactions of platelets and endothelial cells were analyzed in the microvasculature of the inflamed cremaster muscle of CX_3_CR1^GFP/+^ mice by multichannel in vivo microscopy. Panels show results for intravascularly rolling and firmly adherent platelets in venules after 6 h of intrascrotal stimulation with PBS or TNF in dependency of the venular diameter (**A**; mean ± SEM for *n* = 4 per group; #*p* < 0.05 versus PBS). Panel (**B**) shows the adhesion dynamics of platelets, neutrophils, and iMOs during the course of the acute inflammatory response elicited by TNF (mean ± SEM for *n* = 3–4 per group). Interactions of neutrophils and iMOs with endothelial cells were analyzed in the microvasculature of the inflamed cremaster muscle of CX_3_CR1^GFP/+^ mice by multichannel in vivo microscopy. Panels show results for intravascularly rolling and firmly adherent as well as transmigrated leukocytes in venules after 6 h of intrascrotal stimulation with PBS or TNF receiving a platelet-depleting anti-CD42b mAb or isotype control antibodies in dependency of the venular diameter (**C**; mean ± SEM for *n* = 4 per group; #*p* < 0.05 versus PBS; *p < 0.05 versus isotype control).
(TIF)

S3 Fig Interactions of neutrophils and iMOs in the IL-1β- or IFN-γ-stimulated microvasculature. Interactions of neutrophils and iMOs with endothelial cells were analyzed in the microvasculature of the inflamed cremaster muscle of CX_3_CR1^GFP/+^ mice by multichannel in vivo microscopy. Panels show results for intravascularly rolling and firmly adherent as well as transmigrated leukocytes in venules after 6 h of intrascrotal stimulation with PBS, IL-1β (**A**), or IFN-γ (**B**) receiving a platelet-depleting anti-CD42b mAb or isotype control antibodies in dependency of the venular diameter (mean ± SEM for *n* = 4 per group; *p < 0.05 versus isotype control).
(TIF)

S4 Fig Effect of platelets on intraluminal crawling of myeloid leukocytes. Intraluminal crawling of myeloid leukocytes was analyzed by multichannel in vivo microscopy in the microvasculature of the inflamed cremaster muscle of CX_3_CR1^GFP/+^ mice receiving a platelet-depleting anti-CD42b mAb or isotype control antibodies. Panels show results for the proportion of intraluminally crawling neutrophils, iMOs, and ncMOs to total intravascularly adherent neutrophils, iMOs, or ncMOs in venules after 6 h of intrascrotal stimulation with CCL2 (mean ± SEM for *n* = 4 per group).
(TIF)

S5 Fig Neutrophil penetration of the perivenular basement membrane. Using immunostaining and confocal microscopy on tissue whole mounts of the cremaster muscle of WT mice, the transmigration routes of neutrophils through the collagen IV layer of the perivenular basement membrane were analyzed upon stimulation with CCL2. The panel shows quantitative data for the colocalization of Ly-6G^+^ neutrophils and LERs of collagen IV (mean ± SEM for *n* = 3 per group; scale bar: 20 μm).
(TIF)

S6 Fig Adherence of leukocytes to ADP-activated platelets in vitro. Adherence of neutrophils or iMOs to platelets isolated from the peripheral blood of WT mice was measured in vitro by flow cytometry as detailed in Material and Methods. Panel (**A**) shows results for ADP-stimulated or PBS-treated platelets. Panels (**B, C**) show results for ADP-stimulated platelets upon antibody blockade of CD40L/CD154, P-selectin/CD62P, or CD40 (mean ± SEM; *n* = 4–6 per group; #*p* < 0.05, versus unstimulated; *p < 0.05, versus isotype control).
(TIF)

S7 Fig Expression of P-selectin/CD62P on endothelial cells and platelets. Representative multichannel in vivo microscopy images of P-selectin/CD62P expression in CCL2-stimulated cremasteric venular microvessels (scale bar: 10 μm). The panel shows quantitative data for the surface expression of P-selectin/CD62P on endothelial cells, on intravascularly adherent platelets, and on intravascularly adherent platelets capturing a neutrophil or iMO (mean ± SEM; *n* = 4 per group).
(TIF)

S1 Table Platelet and neutrophil depletion. Systemic platelet and neutrophil counts were obtained as detailed in Material and Methods. Quantitative data for animals undergoing 6 h of intrascrotal stimulation with CCL2, TNF, IL-1β, or IFN-γ as well as receiving a platelet-depleting anti-CD42b mAb, a neutrophil-depleting anti-Ly-6G mAb, or isotype control antibodies are shown (mean ± SEM for *n* = 4 per group).
(TIF)

S2 Table Microhemodynamic parameters and systemic leukocyte counts. Systemic leukocyte counts as well as microhemodynamic parameters, including inner vessel diameter, blood flow velocity, and wall shear rate were obtained as detailed in Material and Methods (mean ± SEM for *n* = 3–6 per group).
(TIF)

S1 Video Interactions of neutrophils, monocytes, and platelets in the inflamed microvasculature. Employing multichannel in vivo microscopy, interactions of neutrophils (red), monocytes (green), and platelets (white) were visualized in a postcapillary venule (inner diameter: approximately 25 μm; endothelial junctions in blue) of the cremaster muscle of a CX_3_CR1^GFP/+^ mouse 6 h after stimulation with recombinant murine CCL2.
(MOV)

S2 Video Intravascularly adherent platelets capture crawling leukocytes. Employing multichannel in vivo microscopy, intraluminally crawling neutrophils (red) and monocytes (green) were observed to arrest at sites of intravascularly adherent platelets (white) in a postcapillary venule of the cremaster muscle of a CX_3_CR1^GFP/+^ mouse 6 h after stimulation with recombinant murine CCL2.
(MOV)

S3 Video Rolling neutrophils take up intravascularly adherent platelets. Employing multichannel in vivo microscopy, intravascularly rolling neutrophils (red) were observed to take up intravascularly adherent platelets (white) in a postcapillary venule of the cremaster muscle of a CX_3_CR1^GFP/+^ mouse 6 h after stimulation with recombinant murine CCL2.
(MOV)

S4 Video Transmigration of neutrophils in groups. Employing multichannel in vivo microscopy on the cremaster muscle of a CX_3_CR1^GFP/+^ mouse 6 h after stimulation with recombinant murine CCL2, neutrophils (red) were observed to transmigrate from the microvasculature into the perivascular tissue predominantly in groups.
(MOV)

## Abbreviations

ADP: adenosine diphosphate
iMO: inflammatory monocyte
LER: low expression region
ncMO: non-classical monocyte
PBS: phosphate-buffered saline
SEM: standard error of the mean
TNF: tumor necrosis factor
vWF: von Willebrand factor
