# Supplementary figures and images for: Platelets Guide Leukocytes to Their Sites of Extravasation
Source: PLoS Biol. 2016 May 6;14(5):e1002459. doi: 10.1371/journal.pbio.1002459 (PMC4859536; doi:10.1371/journal.pbio.1002459)

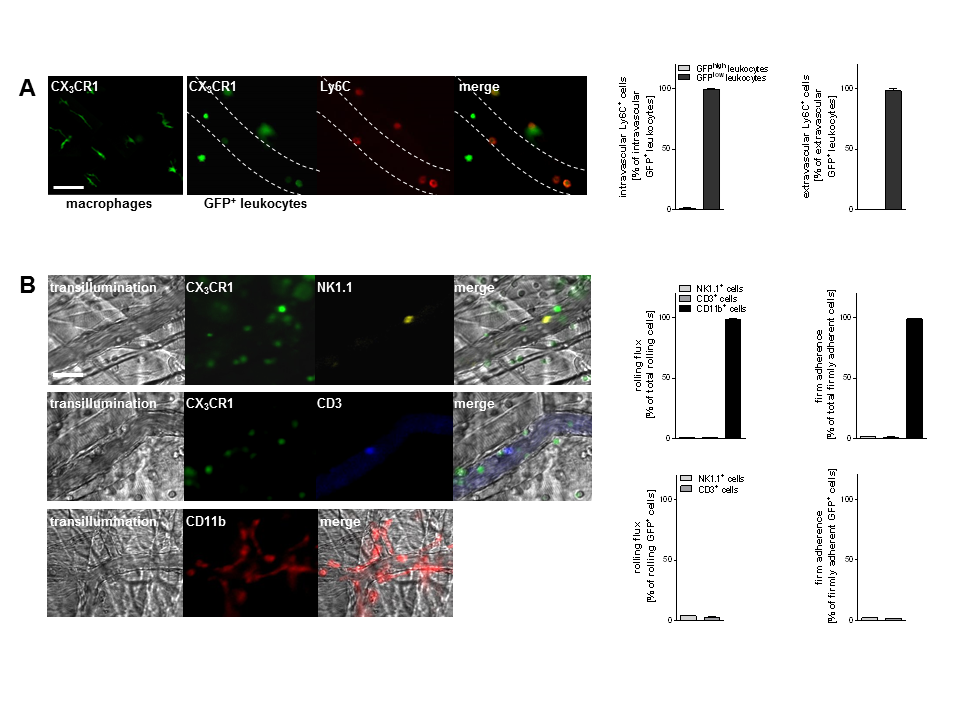

Supplement: S1 Fig — Using multichannel in vivo microscopy on the cremaster muscle of CX3CR1GFP/+ mice, inflammatory/classical monocytes (GFPlow Ly6C+), and ncMOs (GFPhigh Ly6C-) were differentiated by their relative fluorescence intensity of GFP and by in vivo immunostaining for Ly6C, GFP+ tissue macrophages were identified by their morphology. Representative images (A; scale bar: 30 μm) and quantitative data for the expression of Ly6C in intravascular or extravascular GFPhigh and GFPlow leukocytes are shown. NK1.1+ NK cells and CD3+ T cells were identified by in vivo immunostaining for NK1.1 or CD3. Representative images (B; scale bar: 30 μm) and quantitative data for the expression of NK1.1 or CD3 in rolling and adherent total leukocytes or GFP+ leukocytes are shown (mean ± SEM for n = 4 per group). (TIF) [file pbio.1002459.s002.tif]

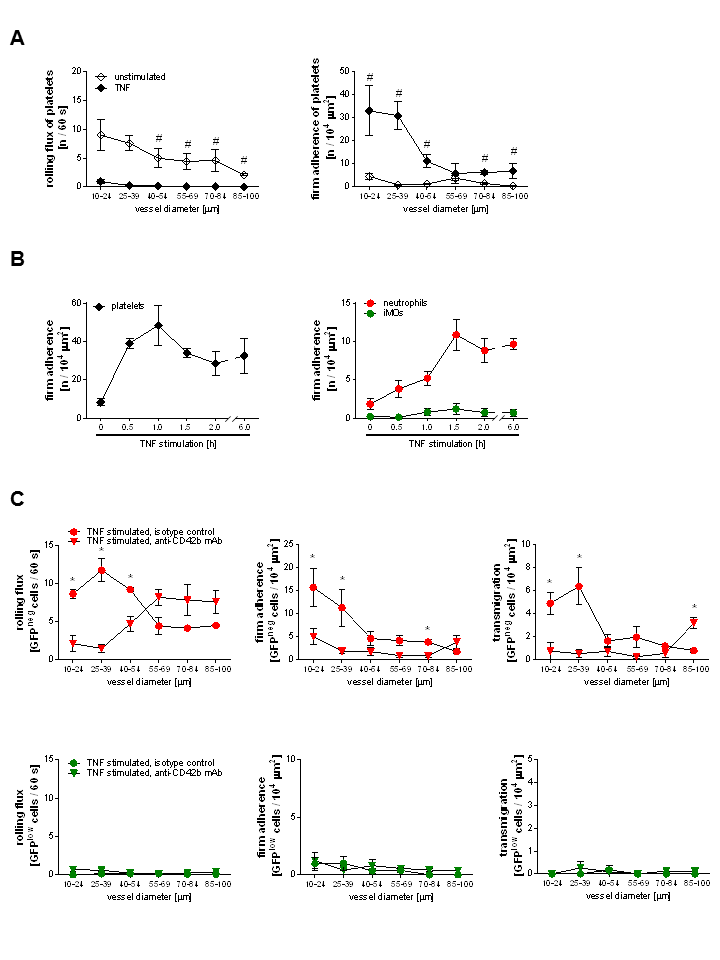

Supplement: S2 Fig — Interactions of platelets and endothelial cells were analyzed in the microvasculature of the inflamed cremaster muscle of CX3CR1GFP/+ mice by multichannel in vivo microscopy. Panels show results for intravascularly rolling and firmly adherent platelets in venules after 6 h of intrascrotal stimulation with PBS or TNF in dependency of the venular diameter (A; mean ± SEM for n = 4 per group; #p < 0.05 versus PBS). Panel (B) shows the adhesion dynamics of platelets, neutrophils, and iMOs during the course of the acute inflammatory response elicited by TNF (mean ± SEM for n = 3–4 per group). Interactions of neutrophils and iMOs with endothelial cells were analyzed in the microvasculature of the inflamed cremaster muscle of CX3CR1GFP/+ mice by multichannel in vivo microscopy. Panels show results for intravascularly rolling and firmly adherent as well as transmigrated leukocytes in venules after 6 h of intrascrotal stimulation with PBS or TNF receiving a platelet-depleting anti-CD42b mAb or isotype control antibodies in dependency of the venular diameter (C; mean ± SEM for n = 4 per group; #p < 0.05 versus PBS; *p < 0.05 versus isotype control). (TIF) [file pbio.1002459.s003.tif]

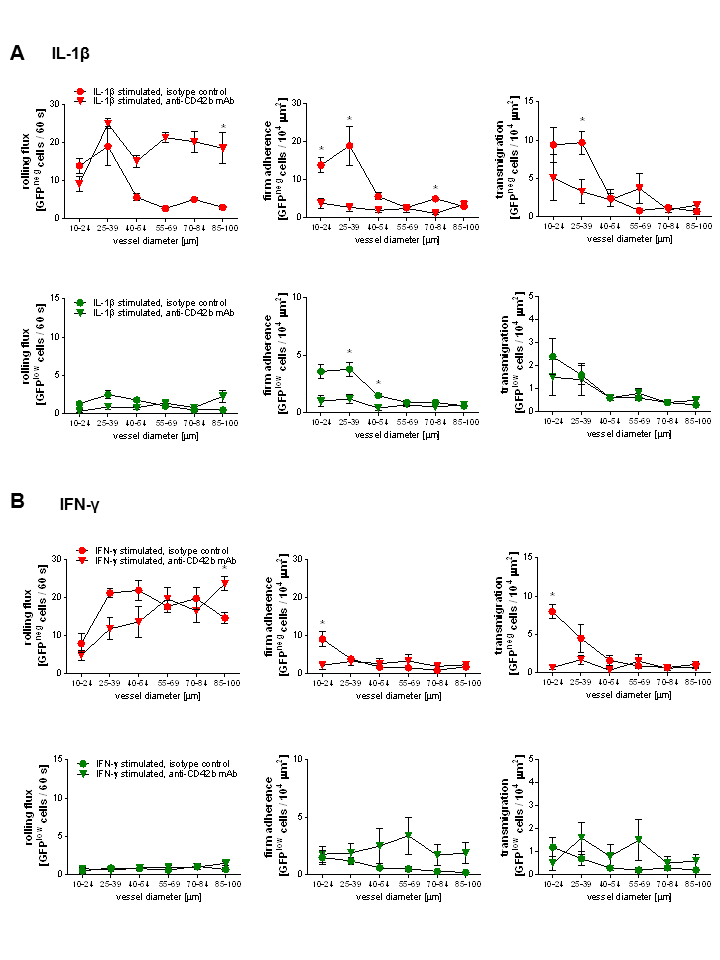

Supplement: S3 Fig — Interactions of neutrophils and iMOs with endothelial cells were analyzed in the microvasculature of the inflamed cremaster muscle of CX3CR1GFP/+ mice by multichannel in vivo microscopy. Panels show results for intravascularly rolling and firmly adherent as well as transmigrated leukocytes in venules after 6 h of intrascrotal stimulation with PBS, IL-1β (A), or IFN-γ (B) receiving a platelet-depleting anti-CD42b mAb or isotype control antibodies in dependency of the venular diameter (mean ± SEM for n = 4 per group; *p < 0.05 versus isotype control). (TIF) [file pbio.1002459.s004.tif]

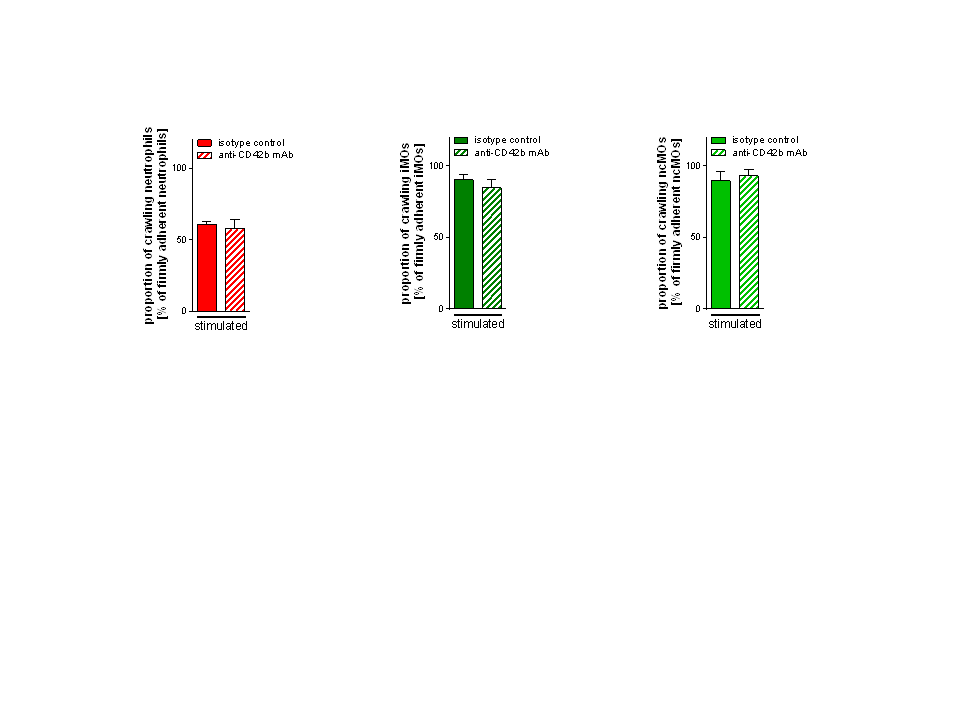

Supplement: S4 Fig — Intraluminal crawling of myeloid leukocytes was analyzed by multichannel in vivo microscopy in the microvasculature of the inflamed cremaster muscle of CX3CR1GFP/+ mice receiving a platelet-depleting anti-CD42b mAb or isotype control antibodies. Panels show results for the proportion of intraluminally crawling neutrophils, iMOs, and ncMOs to total intravascularly adherent neutrophils, iMOs, or ncMOs in venules after 6 h of intrascrotal stimulation with CCL2 (mean ± SEM for n = 4 per group). (TIF) [file pbio.1002459.s005.tif]

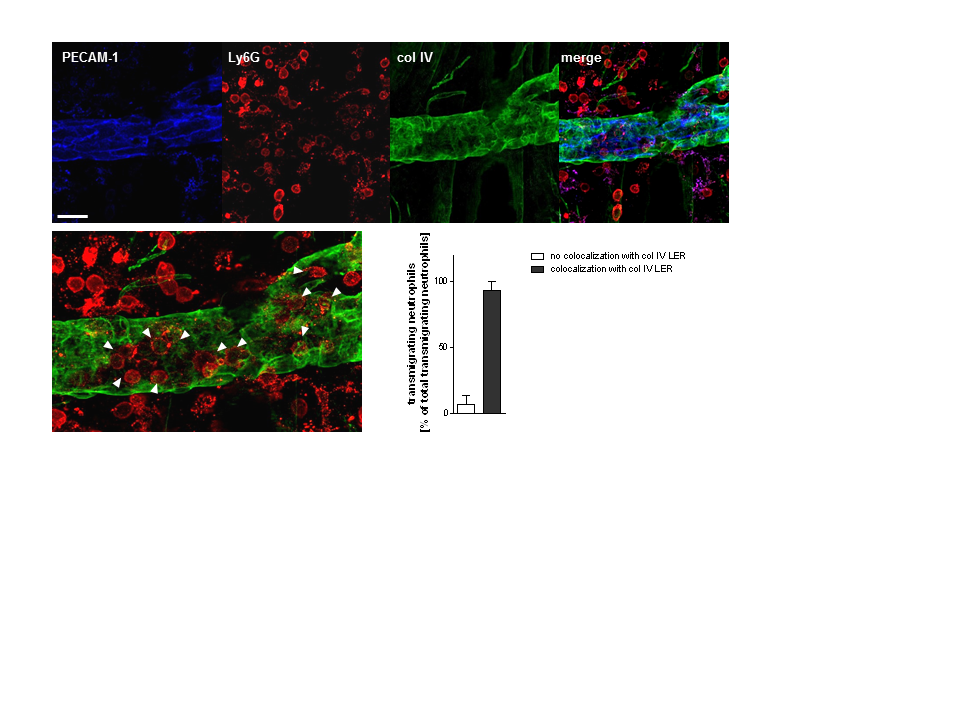

Supplement: S5 Fig — Using immunostaining and confocal microscopy on tissue whole mounts of the cremaster muscle of WT mice, the transmigration routes of neutrophils through the collagen IV layer of the perivenular basement membrane were analyzed upon stimulation with CCL2. The panel shows quantitative data for the colocalization of Ly-6G+ neutrophils and LERs of collagen IV (mean ± SEM for n = 3 per group; scale bar: 20 μm). (TIF) [file pbio.1002459.s006.tif]

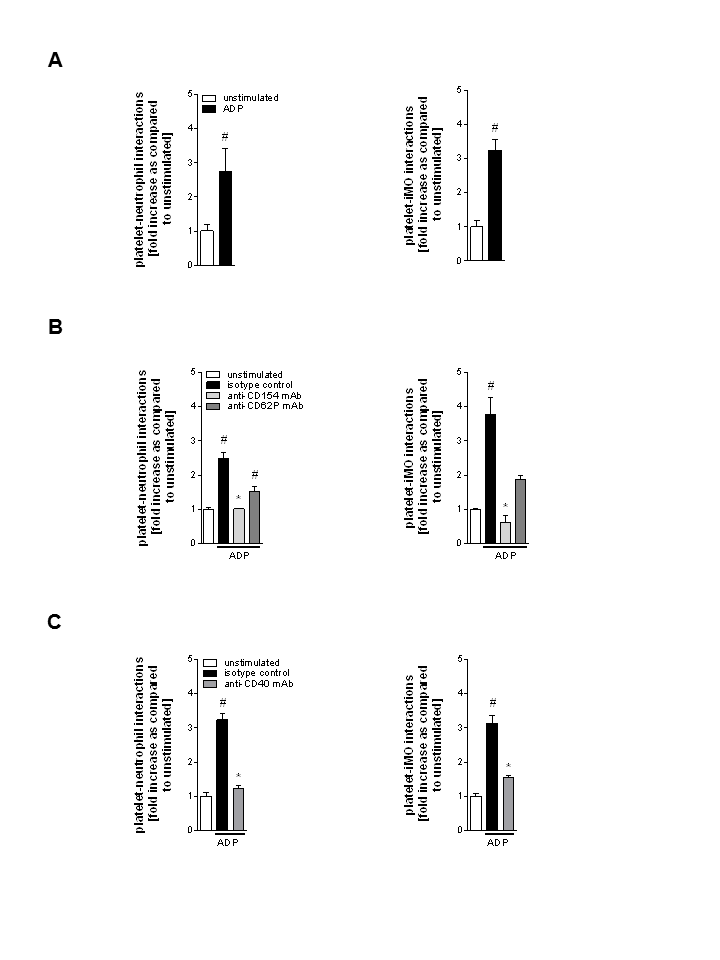

Supplement: S6 Fig — Adherence of neutrophils or iMOs to platelets isolated from the peripheral blood of WT mice was measured in vitro by flow cytometry as detailed in Material and Methods. Panel (A) shows results for ADP-stimulated or PBS-treated platelets. Panels (B, C) show results for ADP-stimulated platelets upon antibody blockade of CD40L/CD154, P-selectin/CD62P, or CD40 (mean ± SEM; n = 4–6 per group; #p < 0.05, versus unstimulated; *p < 0.05, versus isotype control). (TIF) [file pbio.1002459.s007.tif]

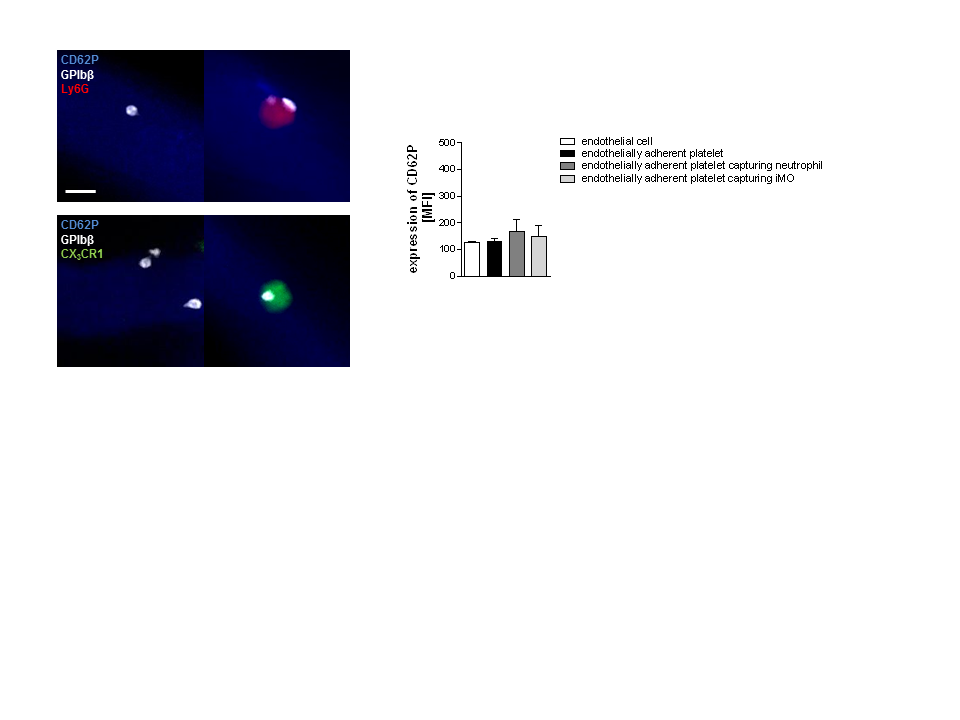

Supplement: S7 Fig — Representative multichannel in vivo microscopy images of P-selectin/CD62P expression in CCL2-stimulated cremasteric venular microvessels (scale bar: 10 μm). The panel shows quantitative data for the surface expression of P-selectin/CD62P on endothelial cells, on intravascularly adherent platelets, and on intravascularly adherent platelets capturing a neutrophil or iMO (mean ± SEM; n = 4 per group). (TIF) [file pbio.1002459.s008.tif]

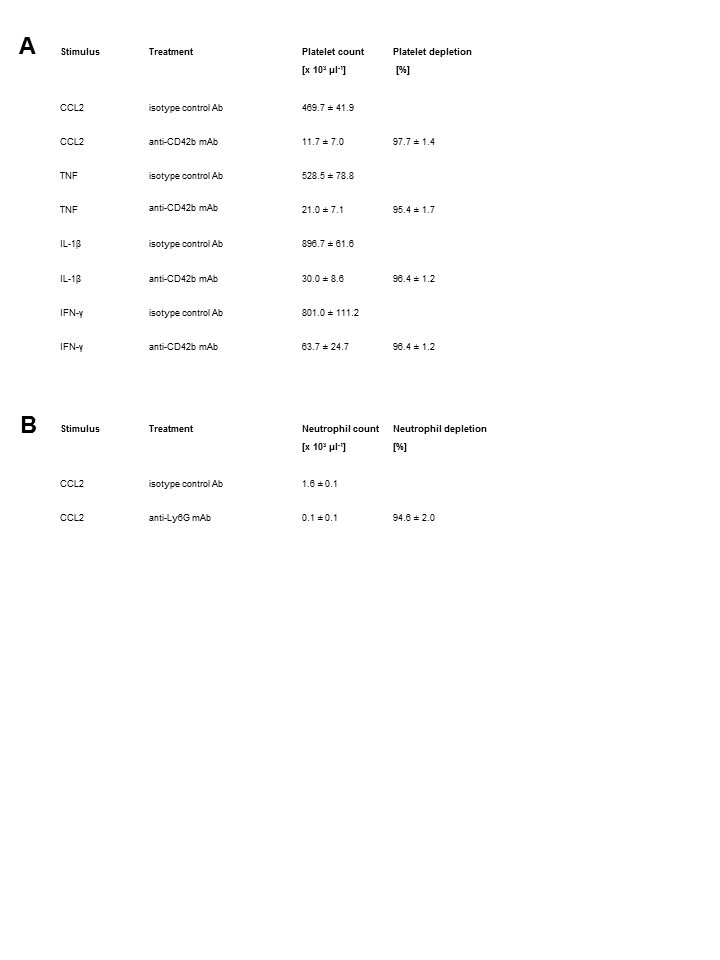

Supplement: S1 Table — Systemic platelet and neutrophil counts were obtained as detailed in Material and Methods. Quantitative data for animals undergoing 6 h of intrascrotal stimulation with CCL2, TNF, IL-1β, or IFN-γ as well as receiving a platelet-depleting anti-CD42b mAb, a neutrophil-depleting anti-Ly-6G mAb, or isotype control antibodies are shown (mean ± SEM for n = 4 per group). (TIF) [file pbio.1002459.s009.tif]

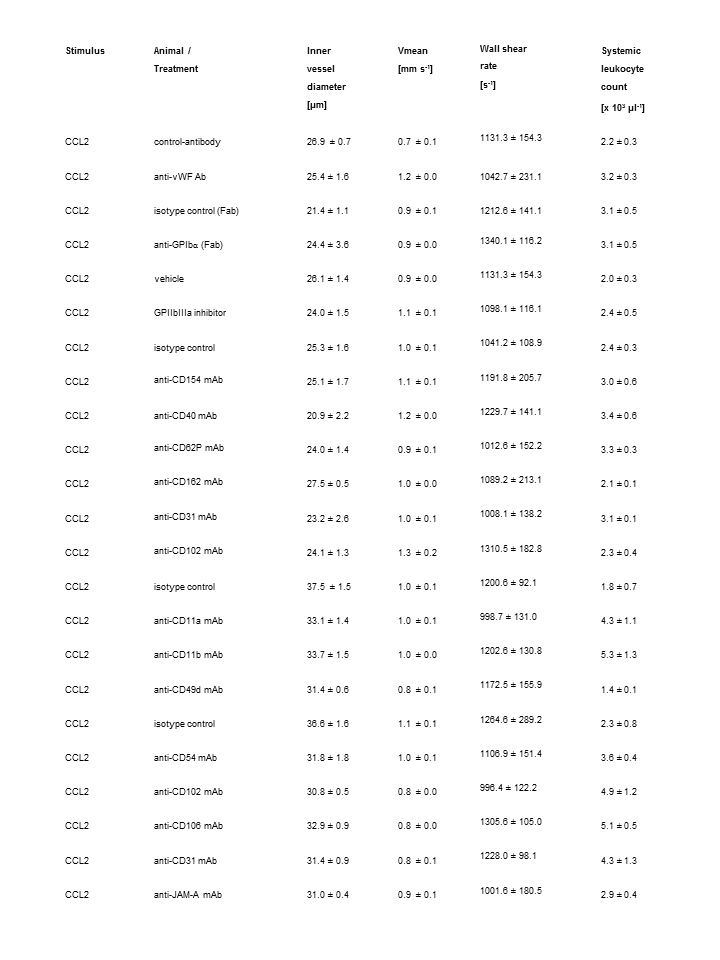

Supplement: S2 Table — Systemic leukocyte counts as well as microhemodynamic parameters, including inner vessel diameter, blood flow velocity, and wall shear rate were obtained as detailed in Material and Methods (mean ± SEM for n = 3–6 per group). (TIF) [file pbio.1002459.s010.tif]
